# Supplementary material for: Mitigating Biases in CORD-19 for Analyzing COVID-19 Literature
Source: Front Res Metr Anal. 2020 Nov 23;5:596624. doi: 10.3389/frma.2020.596624 (PMC8025972; doi:10.3389/frma.2020.596624)
Supplement: Supplementary file 1 [file DataSheet1.zip › Cord-19 Expansion Figures.pptx]

## Slide 1
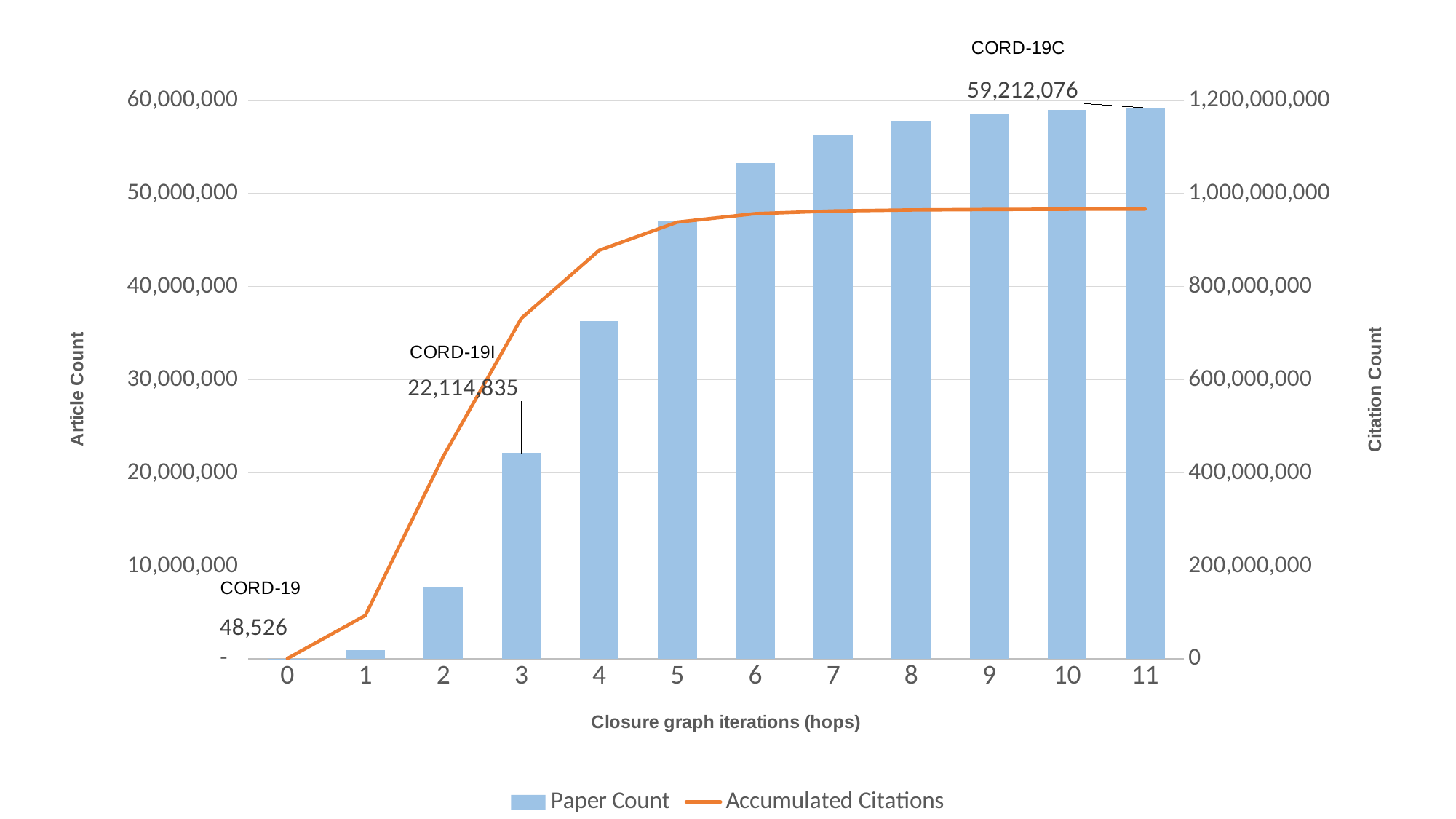

### Chart
| Category | | |
|---|---|---|
| 0 | 48526.0 | 971840.0 |
| 1 | 974807.0 | 93645312.0 |
| 2 | 7777366.0 | 435395489.0 |
| 3 | 22114835.0 | 731920368.0 |
| 4 | 36287938.0 | 878335449.0 |
| 5 | 47013914.0 | 938708765.0 |
| 6 | 53254829.0 | 956964267.0 |
| 7 | 56320362.0 | 962685184.0 |
| 8 | 57798530.0 | 964894612.0 |
| 9 | 58552754.0 | 965903418.0 |
| 10 | 58967628.0 | 966416544.0 |
| 11 | 59212076.0 | 966676270.0 |

## Slide 2
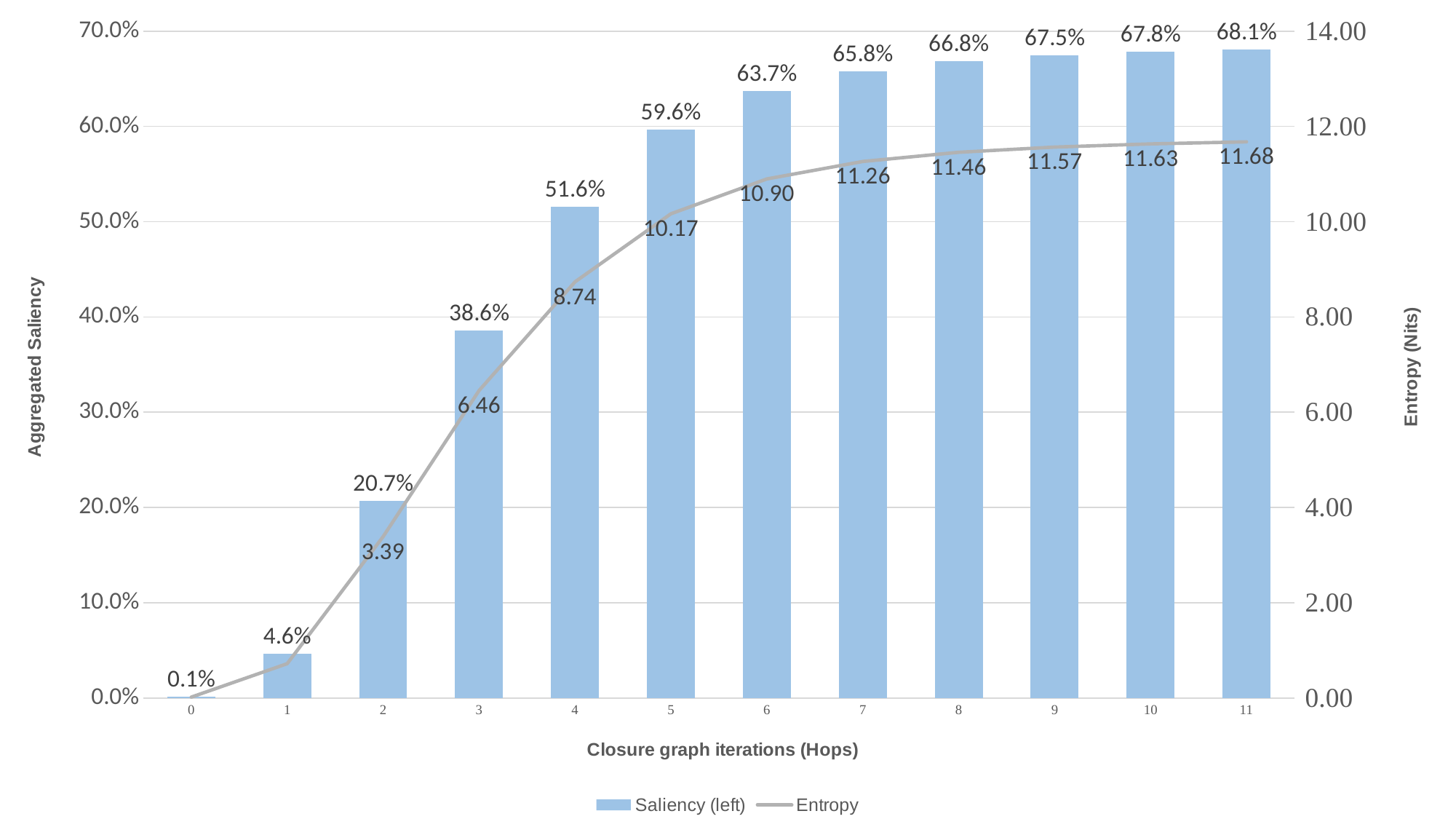

### Chart
| Category | Saliency (left) | Entropy |
|---|---|---|
| 0 | 0.00107971603621735 | 0.018126589367863773 |
| 1 | 0.04626365553415232 | 0.7210117978210053 |
| 2 | 0.20697422039758823 | 3.3876740840913113 |
| 3 | 0.3855790748985589 | 6.455424549388589 |
| 4 | 0.5159573145944508 | 8.737779554735393 |
| 5 | 0.5963447019919585 | 10.167352912411266 |
| 6 | 0.6371217979651569 | 10.89791354818471 |
| 7 | 0.6576105038143218 | 11.264841047471617 |
| 8 | 0.6684304092552393 | 11.45813770435322 |
| 9 | 0.6745774652267376 | 11.567405946252647 |
| 10 | 0.6783179104043772 | 11.633634805486954 |
| 11 | 0.6807629635638364 | 11.676752864081175 |

## Slide 3
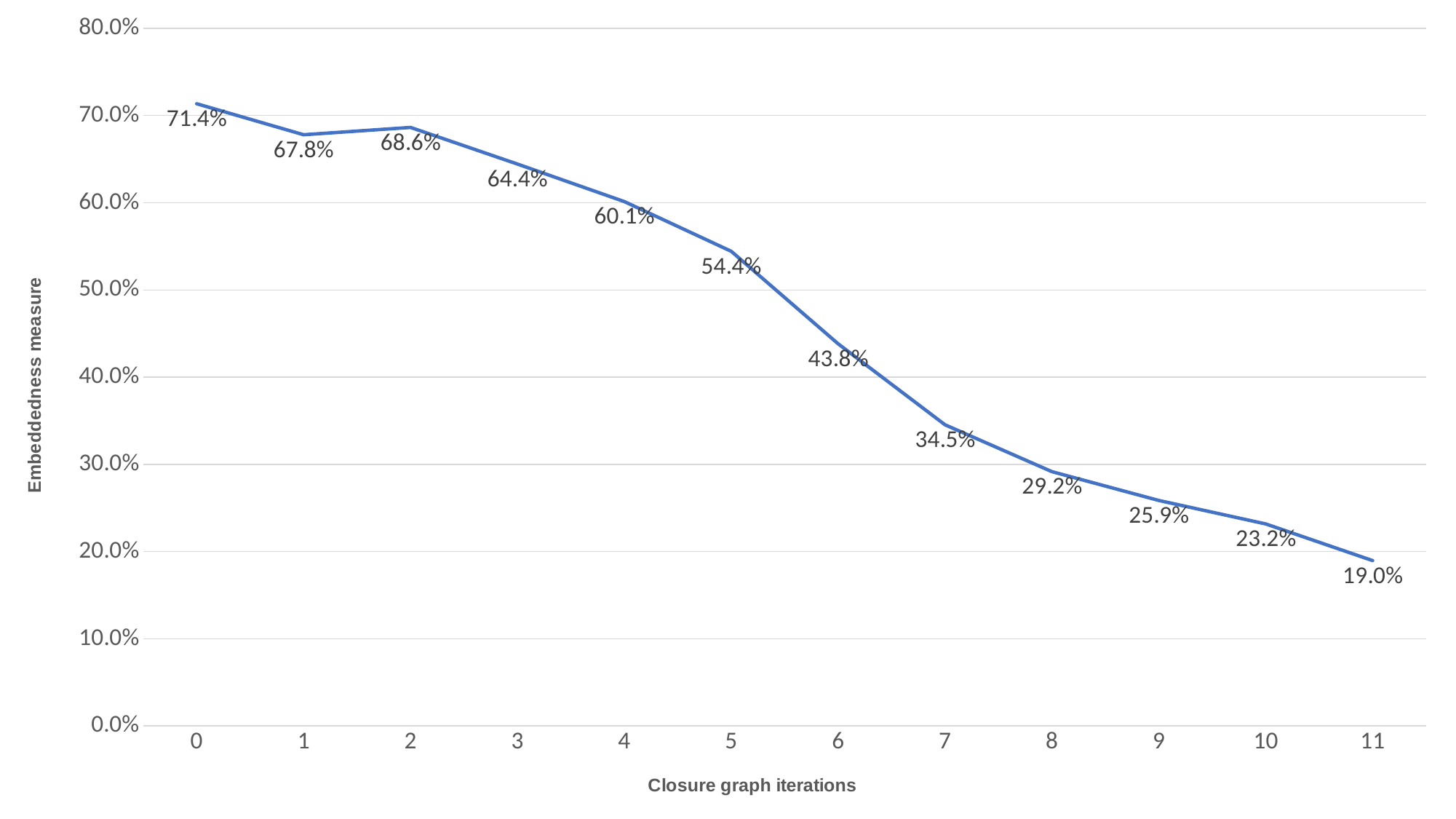

### Chart
| Category | Specialization |
|---|---|
| 0 | 0.7135258163396413 |
| 1 | 0.6779688603237851 |
| 2 | 0.6863614875516564 |
| 3 | 0.644355003160106 |
| 4 | 0.6012831465184886 |
| 5 | 0.5442960478983785 |
| 6 | 0.4382198111580202 |
| 7 | 0.3452815186668379 |
| 8 | 0.29155607870357236 |
| 9 | 0.2585463568707876 |
| 10 | 0.23163271800969368 |
| 11 | 0.18955141313692606 |

## Slide 4
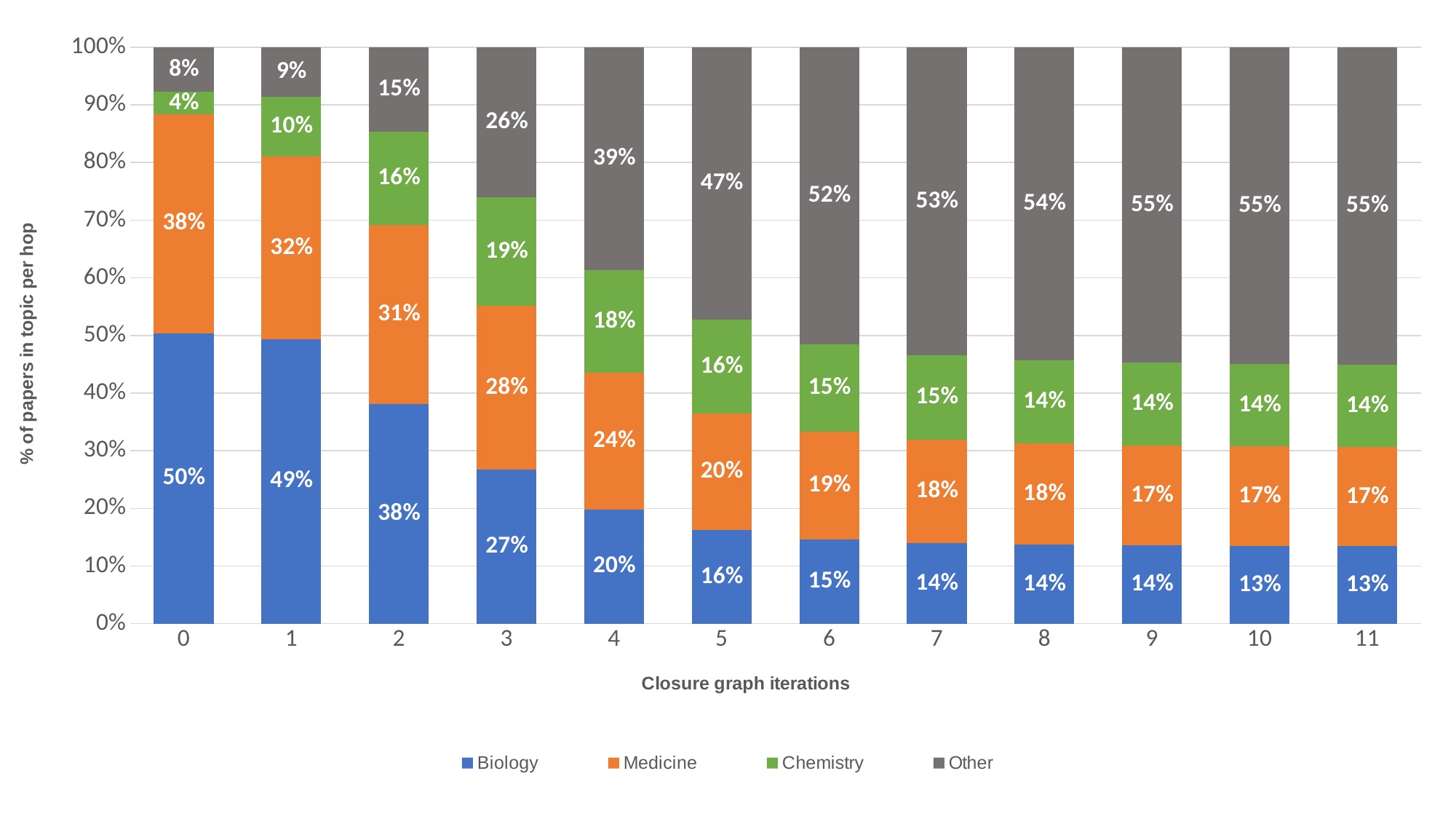

### Chart
| Category | Biology | Medicine | Chemistry | Other |
|---|---|---|---|---|
| 0 | 0.5039875878149626 | 0.3796208903118212 | 0.038724162936501196 | 0.07766735893671509 |
| 1 | 0.49367920745353855 | 0.3161689364399185 | 0.10448976164096993 | 0.08566209446557296 |
| 2 | 0.3812143744774065 | 0.3099225226687767 | 0.16232296590189418 | 0.14654013695192258 |
| 3 | 0.2672429390173435 | 0.2839578241523529 | 0.18854999588231336 | 0.2602492409479903 |
| 4 | 0.1977830216677426 | 0.2371388798393297 | 0.1786450689080788 | 0.3864330295848489 |
| 5 | 0.16213097364567378 | 0.20299054641041778 | 0.16181237616714034 | 0.4730661037767681 |
| 6 | 0.1464959160594832 | 0.18615384757469625 | 0.151840841195248 | 0.5155093951705726 |
| 7 | 0.13989991268585764 | 0.1787748123225944 | 0.14711125964396904 | 0.534214015347579 |
| 8 | 0.136901928539094 | 0.17537898929700105 | 0.14487712863178664 | 0.5428419535321183 |
| 9 | 0.1354018582143774 | 0.1736642491583822 | 0.14373883784049488 | 0.5471950547867456 |
| 10 | 0.1345805493016035 | 0.1727249364373193 | 0.14310867026477805 | 0.5495858439962991 |
| 11 | 0.13409964794250873 | 0.17214619192150743 | 0.14273285699093782 | 0.551021303145046 |

## Slide 5
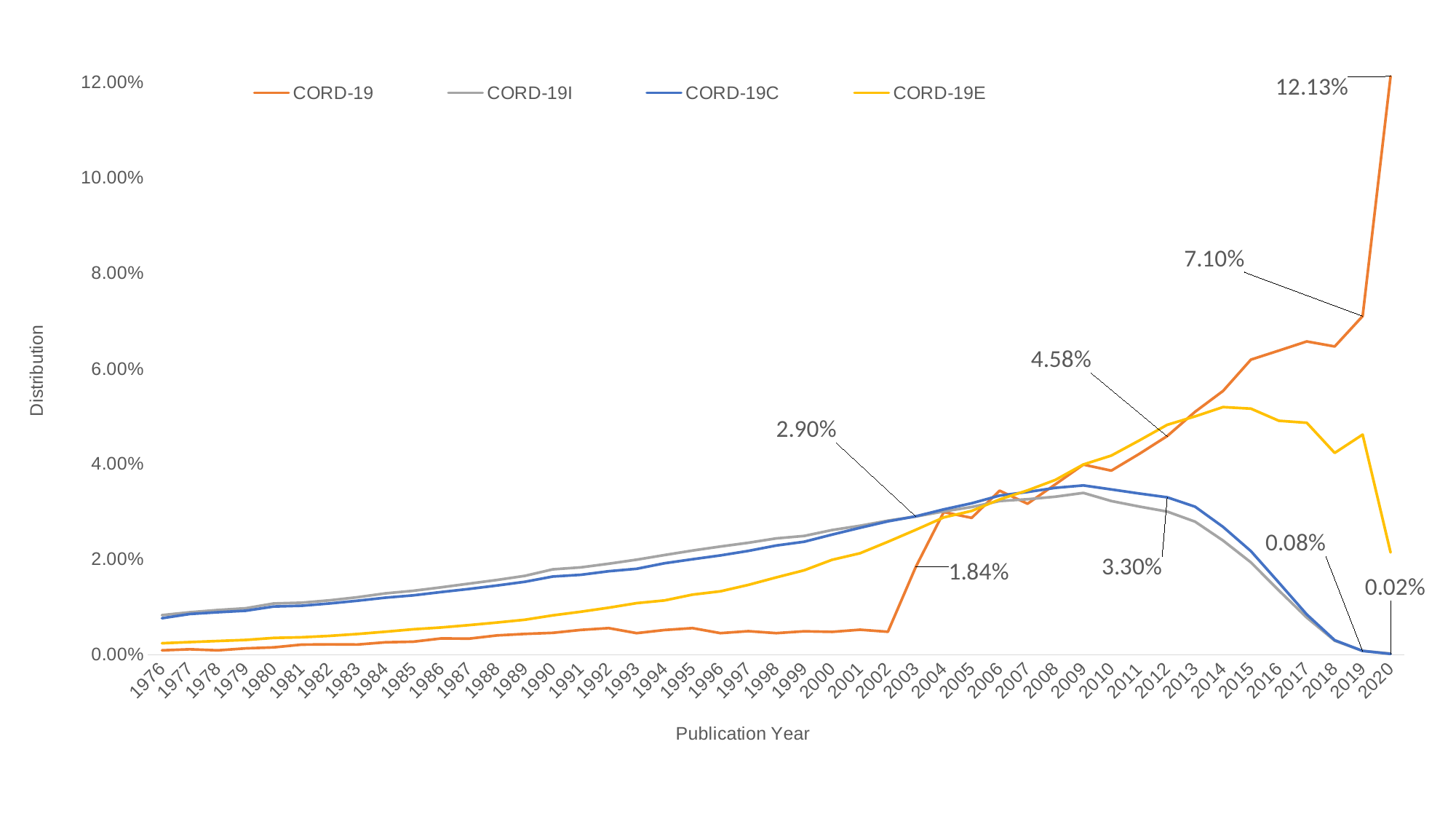

### Chart
| Category | | | | |
|---|---|---|---|---|
| 1976 | 0.0009067304125623377 | 0.008286753773908133 | 0.007643448457430525 | 0.002391431121850815 |
| 1977 | 0.0011334130157029223 | 0.008896681382585987 | 0.008527964855512353 | 0.002636723435281684 |
| 1978 | 0.0009067304125623377 | 0.009359452553802144 | 0.008896707572045722 | 0.002860392183892201 |
| 1979 | 0.001318880600090673 | 0.009733654288688358 | 0.009214160024307229 | 0.003087439614505898 |
| 1980 | 0.0015249556938548407 | 0.010736632883792462 | 0.010097257791437842 | 0.00351315354690658 |
| 1981 | 0.0021019659563945104 | 0.010892855701015376 | 0.01025778263895317 | 0.003646949354232509 |
| 1982 | 0.0021637884845237603 | 0.011390486964626486 | 0.010730592068836297 | 0.003933461588102174 |
| 1983 | 0.002122573465770927 | 0.01204312852050764 | 0.011310085923943445 | 0.004328767382474236 |
| 1984 | 0.0025965461814285128 | 0.012850697696502966 | 0.011951324002355806 | 0.004812594645329614 |
| 1985 | 0.002720191237687013 | 0.013391125579473253 | 0.012439721484238579 | 0.005327505782614248 |
| 1986 | 0.0034002390471087663 | 0.014124014241326031 | 0.013131743173011655 | 0.005705242430569774 |
| 1987 | 0.003359024028355933 | 0.014900333342399713 | 0.013777912682825853 | 0.006196502793832148 |
| 1988 | 0.004039071837777687 | 0.015661688783259593 | 0.01450621079163711 | 0.006742497805546039 |
| 1989 | 0.004348184478423937 | 0.016536112175974407 | 0.015290764373049477 | 0.007327009792096182 |
| 1990 | 0.004574867081564522 | 0.017890557663098453 | 0.016385508367330232 | 0.008246011296961146 |
| 1991 | 0.0051930923628570255 | 0.01830970549339666 | 0.01674543530580963 | 0.009004863274875378 |
| 1992 | 0.005564027531632527 | 0.019073155295538765 | 0.017500434075738376 | 0.009850885248471653 |
| 1993 | 0.0045130445534352715 | 0.0199237691073609 | 0.018010381237308637 | 0.010810430937374778 |
| 1994 | 0.005172484853480608 | 0.020899355661484306 | 0.01917374305962251 | 0.011370616413502024 |
| 1995 | 0.005564027531632527 | 0.02183600363018948 | 0.020015362759893823 | 0.012590996353050646 |
| 1996 | 0.0045130445534352715 | 0.022684467965958802 | 0.020815521281769545 | 0.013286329109305092 |
| 1997 | 0.004925194740963607 | 0.023468778708767297 | 0.021762524995559602 | 0.014630368810170102 |
| 1998 | 0.0045130445534352715 | 0.024385475130520733 | 0.022891637013479883 | 0.016200780205248174 |
| 1999 | 0.004904587231587191 | 0.02489892433328971 | 0.02367985539151622 | 0.017694833387054378 |
| 2000 | 0.00478094217532869 | 0.026130188308156492 | 0.02518269222276282 | 0.019891652425522023 |
| 2001 | 0.005234307381609859 | 0.027033216266804872 | 0.026601373839367774 | 0.02126677600081629 |
| 2002 | 0.004801549684705106 | 0.0281058599319283 | 0.027959712975155027 | 0.02367037037787855 |
| 2003 | 0.01842311338251659 | 0.02897050045286429 | 0.029018957418728026 | 0.02619154288865148 |
| 2004 | 0.02990149610518073 | 0.03003688859152633 | 0.030468308485421126 | 0.028789073612696277 |
| 2005 | 0.02868565305197214 | 0.030967088132073068 | 0.03174781227263378 | 0.03014933098717655 |
| 2006 | 0.03439393314923958 | 0.03221744937494752 | 0.03336422402229602 | 0.032554952573440724 |
| 2007 | 0.03165313440217615 | 0.032598953816936234 | 0.034110423902634496 | 0.034459853486833614 |
| 2008 | 0.035733421258706675 | 0.033131886091106975 | 0.034991579496439044 | 0.03665802399810253 |
| 2009 | 0.03985492313399003 | 0.033926751312482475 | 0.035495261037974776 | 0.03988804399314263 |
| 2010 | 0.038597865062028607 | 0.03221830365389158 | 0.03465749190742818 | 0.04175240072249736 |
| 2011 | 0.042101141656019456 | 0.031068885113343388 | 0.033797075347053285 | 0.04491146839547067 |
| 2012 | 0.04583110085315089 | 0.030007953336969255 | 0.033002152248926095 | 0.048194871566161014 |
| 2013 | 0.05094176317850225 | 0.027891987064673572 | 0.031034713781156957 | 0.049976788454638155 |
| 2014 | 0.0553105551663026 | 0.023937584947362502 | 0.026802312780528994 | 0.051929666652476204 |
| 2015 | 0.06188435065737955 | 0.019350933739286688 | 0.021714679977883542 | 0.051603961707369646 |
| 2016 | 0.0637802415200099 | 0.013464841585130564 | 0.015119498462617816 | 0.0490523810585681 |
| 2017 | 0.06569673989201665 | 0.007775288702880212 | 0.008434774312788568 | 0.04862869433536933 |
| 2018 | 0.0646457569138194 | 0.0029278619577819205 | 0.003038200843588895 | 0.04230988325302206 |
| 2019 | 0.07101347731113218 | 0.0007878105307406354 | 0.0007869686317221722 | 0.046146714535833286 |
| 2020 | 0.12129580018958909 | 0.00022718308435097935 | 0.000159342655055983 | 0.021433007155372746 |

## Slide 6
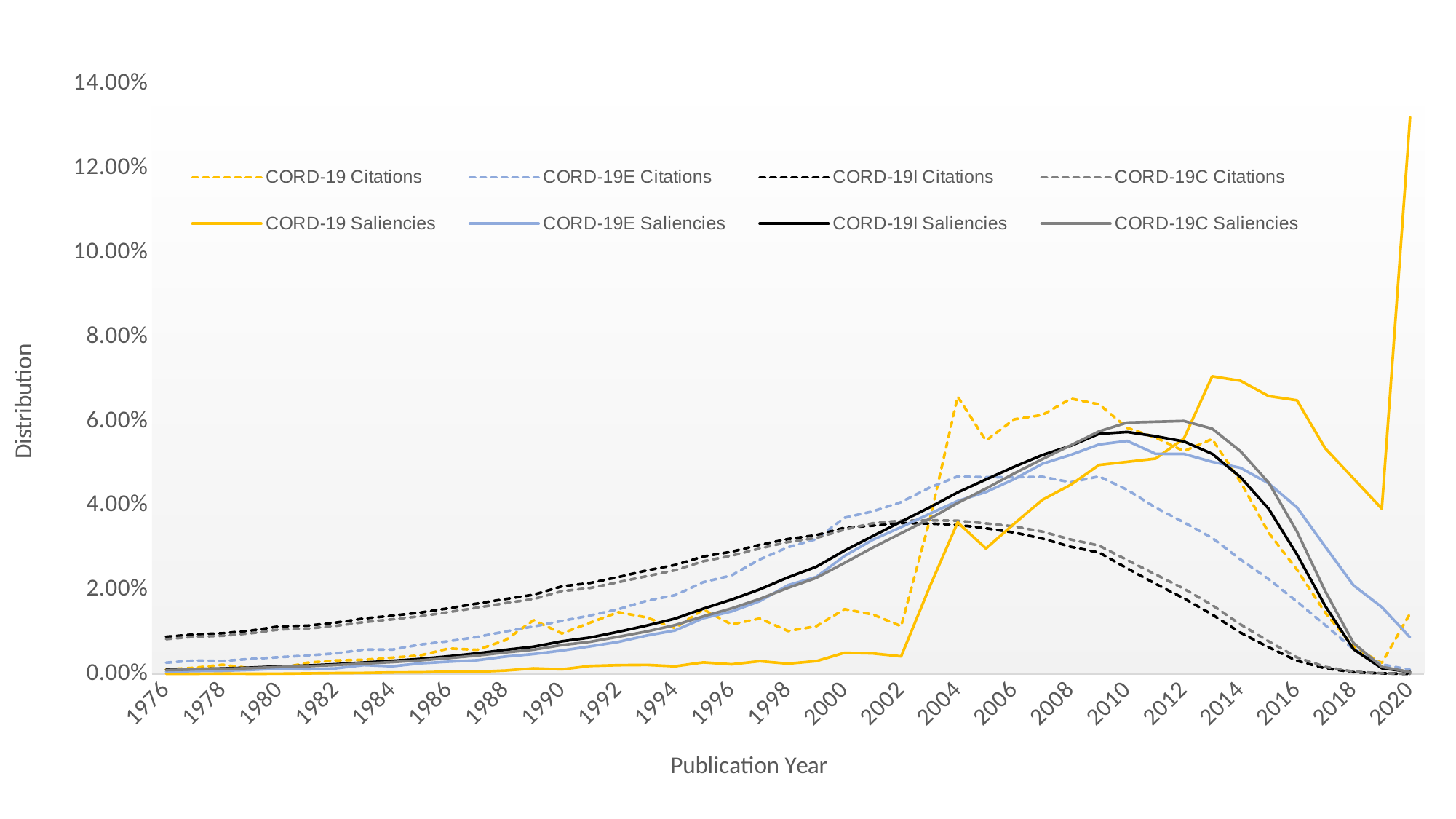

### Chart
| Category | | | | | | | | |
|---|---|---|---|---|---|---|---|---|
| 1976 | 0.001102033256503128 | 0.0027 | 0.008846789095638668 | 0.008291039566121487 | 4.8167439487040674e-05 | 0.000546609 | 0.0010181892851517525 | 0.0009320633031969384 |
| 1977 | 0.0014724646032268686 | 0.0032 | 0.00939475099764942 | 0.008844332428299085 | 7.852367900508086e-05 | 0.000722669 | 0.0011906091622893035 | 0.0011188860632079206 |
| 1978 | 0.002206124464932499 | 0.0031 | 0.009672125023371992 | 0.009087348671200502 | 0.00010812710666056177 | 0.000767317 | 0.0013241363418964045 | 0.001235703447071776 |
| 1979 | 0.0013757408626934476 | 0.0036 | 0.010312470088473183 | 0.009672024329761946 | 7.363857672576567e-05 | 0.000981458 | 0.0015320625744954556 | 0.0014121171659434756 |
| 1980 | 0.0013047415212380639 | 0.004 | 0.01131538717594726 | 0.010586809516993088 | 8.40409130224497e-05 | 0.001304317 | 0.0018198803238843385 | 0.0016748762704999302 |
| 1981 | 0.0026866562397102403 | 0.0044 | 0.011481429736396823 | 0.010783090355107302 | 0.0001612510159690931 | 0.001139893 | 0.0020218537152830087 | 0.0018648274852992401 |
| 1982 | 0.0032258396443859074 | 0.0049 | 0.012204578696113938 | 0.011442361835511351 | 0.00022604236259107755 | 0.001355949 | 0.002335714487521879 | 0.002145512239077124 |
| 1983 | 0.0033812150148172537 | 0.0058 | 0.013198643040700998 | 0.012328593509020408 | 0.00026613449846281596 | 0.002125706 | 0.0027392677986095874 | 0.002495813576175022 |
| 1984 | 0.0038854132367467896 | 0.0058 | 0.013851546415219136 | 0.01298869256753393 | 0.00037919633940509425 | 0.001840162 | 0.0031243472749912407 | 0.0028349895546245047 |
| 1985 | 0.0044317994731643065 | 0.007 | 0.014602708084940777 | 0.013720187824024309 | 0.00041821364901377904 | 0.002549245 | 0.003604207674299182 | 0.0032566587986571937 |
| 1986 | 0.006070958182416859 | 0.0078 | 0.015626109475544218 | 0.014700404440008282 | 0.0005637867240855944 | 0.002929158 | 0.00421666525957082 | 0.0037902675511901456 |
| 1987 | 0.0057427148501810995 | 0.0088 | 0.01673672254001686 | 0.015758590293103596 | 0.0005449538291017758 | 0.003273604 | 0.004910303079689944 | 0.004394844474937607 |
| 1988 | 0.008035273296015805 | 0.0101 | 0.01779368140381917 | 0.016835048036383436 | 0.0008272366860913168 | 0.004144831 | 0.005734483053137127 | 0.005125743507447089 |
| 1989 | 0.012782968389858413 | 0.0113 | 0.018827713236729427 | 0.01780403234847659 | 0.0013489196221790779 | 0.004744246 | 0.0064890869727628675 | 0.005787865380390668 |
| 1990 | 0.009633272966743496 | 0.0126 | 0.020800048575386365 | 0.019660785560417147 | 0.001118191730698027 | 0.005577266 | 0.007766316707408634 | 0.006877207519859588 |
| 1991 | 0.012194394138952914 | 0.0139 | 0.021606283172909833 | 0.02041955779139575 | 0.0019254519476609349 | 0.006559424 | 0.008669678116477663 | 0.007658878585620802 |
| 1992 | 0.014683486993743826 | 0.0154 | 0.02300404546466123 | 0.021807060767120943 | 0.002108286883112635 | 0.007632901 | 0.01003491254835339 | 0.008845510982788967 |
| 1993 | 0.013419904511030622 | 0.0174 | 0.024587004563305787 | 0.023229584779493752 | 0.002166481918904955 | 0.009135681 | 0.011506608814916218 | 0.010102750425493731 |
| 1994 | 0.01092360882449786 | 0.0187 | 0.025870168884977396 | 0.024621008406512573 | 0.0018362031752875542 | 0.010347406 | 0.013153781626185634 | 0.011615229877011213 |
| 1995 | 0.01533071287454725 | 0.0218 | 0.02789698935219116 | 0.026772420804330923 | 0.0027695718524836654 | 0.013243275 | 0.015522765711965272 | 0.013691626951379848 |
| 1996 | 0.011781774777741193 | 0.0234 | 0.02902718285331423 | 0.02807357531458636 | 0.0023107153719438174 | 0.014860454 | 0.017667396721591722 | 0.015581254485355949 |
| 1997 | 0.013197645702996377 | 0.0272 | 0.03065507355507372 | 0.02978614269593138 | 0.00304056276918508 | 0.017300858 | 0.02008509360392822 | 0.017832554417127854 |
| 1998 | 0.010202296674349687 | 0.0301 | 0.032031353286097186 | 0.03133105516596885 | 0.0024764577343744077 | 0.021086728 | 0.022948176016687652 | 0.020447666393865544 |
| 1999 | 0.011351662825156403 | 0.032 | 0.03296098425018524 | 0.03230131498282323 | 0.003061749009837572 | 0.023038379 | 0.025458923306022334 | 0.022817930226606276 |
| 2000 | 0.015401712216002634 | 0.0371 | 0.03472973224547339 | 0.034277730817030366 | 0.0050508603267636395 | 0.02802387 | 0.029289636423765843 | 0.026376490713169082 |
| 2001 | 0.014121666117879487 | 0.0386 | 0.035212615761000966 | 0.03578741535589285 | 0.0048883188442193784 | 0.031875651 | 0.032748639756082686 | 0.03001840701402223 |
| 2002 | 0.011367097464603227 | 0.0408 | 0.03581775722898359 | 0.03640725067043525 | 0.004196138887330364 | 0.034867085 | 0.036179346388380856 | 0.03341823505540351 |
| 2003 | 0.03619114257490945 | 0.0442 | 0.03570440064514385 | 0.036490812266621354 | 0.020542027074657417 | 0.038005448 | 0.039522610218429476 | 0.036807715062317954 |
| 2004 | 0.06583902700032927 | 0.0469 | 0.03539572309174583 | 0.03639275357090256 | 0.03599293422559561 | 0.041058341 | 0.04309129819241192 | 0.040591654298121886 |
| 2005 | 0.05538051531116233 | 0.0467 | 0.0345897073099997 | 0.03575197121993677 | 0.029776560362434323 | 0.043187694 | 0.046162002617690844 | 0.04400786886166485 |
| 2006 | 0.06044410602568324 | 0.0467 | 0.03358433748358358 | 0.03502811347492419 | 0.035698780923822004 | 0.046250072 | 0.04917760035654166 | 0.04759579768797589 |
| 2007 | 0.06148234277247284 | 0.0468 | 0.03214104577829289 | 0.033786140109749785 | 0.041360321828386316 | 0.049905195 | 0.05198400827613807 | 0.051022041861135126 |
| 2008 | 0.06535643727362529 | 0.0455 | 0.030192518454311264 | 0.03193595683352581 | 0.04491794552744233 | 0.052008657 | 0.05416287572411982 | 0.054274766688599936 |
| 2009 | 0.064007449785973 | 0.0469 | 0.028839271586647317 | 0.03047732248481879 | 0.04960498250070572 | 0.054476961 | 0.0570041277336844 | 0.05758678853290938 |
| 2010 | 0.058405704642739545 | 0.0437 | 0.025054608158800257 | 0.02703769889837716 | 0.05035223981138109 | 0.055312085 | 0.05743721891813872 | 0.059672762922222115 |
| 2011 | 0.05612137800460981 | 0.0395 | 0.02140673319515818 | 0.02363311290014533 | 0.0511198686339233 | 0.052248781 | 0.05641981720322404 | 0.05986500053100505 |
| 2012 | 0.05289142245637142 | 0.036 | 0.017972993067419906 | 0.020241041951874005 | 0.055885796820040176 | 0.05223235 | 0.05519402834098625 | 0.0600473968163737 |
| 2013 | 0.055765352321369775 | 0.0323 | 0.01413046073239394 | 0.0163751235709892 | 0.07066582846821035 | 0.050340676 | 0.05224998877795851 | 0.0582140262233653 |
| 2014 | 0.04565154757984854 | 0.0272 | 0.009829806818891425 | 0.011786799844673934 | 0.0695991773161377 | 0.04892457 | 0.046726549562387705 | 0.052873880561270535 |
| 2015 | 0.033517863022719786 | 0.0225 | 0.0063521462171531005 | 0.007752382422809915 | 0.06595291900035122 | 0.045232771 | 0.03919414654623463 | 0.04538872633926262 |
| 2016 | 0.024764364504445177 | 0.0172 | 0.0031476535156396554 | 0.00395621749735466 | 0.06496092420246666 | 0.039545966 | 0.028370427597862742 | 0.03381040927390757 |
| 2017 | 0.014580589397431676 | 0.0116 | 0.0013643764387700488 | 0.0017081491487130709 | 0.05354922397357098 | 0.030229369 | 0.01616608023327509 | 0.01964647800887131 |
| 2018 | 0.00643521567336187 | 0.0059 | 0.00044562762664117215 | 0.0005753995424268909 | 0.04638558726766933 | 0.021041588 | 0.0058941883043556525 | 0.007362670480420214 |
| 2019 | 0.0026115409944023706 | 0.0023 | 0.00015366159192405256 | 0.00019242535859670683 | 0.039231369849983974 | 0.015890305 | 0.0013785507857272026 | 0.0017228770158445067 |
| 2020 | 0.014381997036549226 | 0.001 | 4.3212540602787036e-05 | 3.9976160578084e-05 | 0.13215724288040048 | 0.008683208 | 0.0005281240614413945 | 0.0004396620883011325 |

## Slide 7
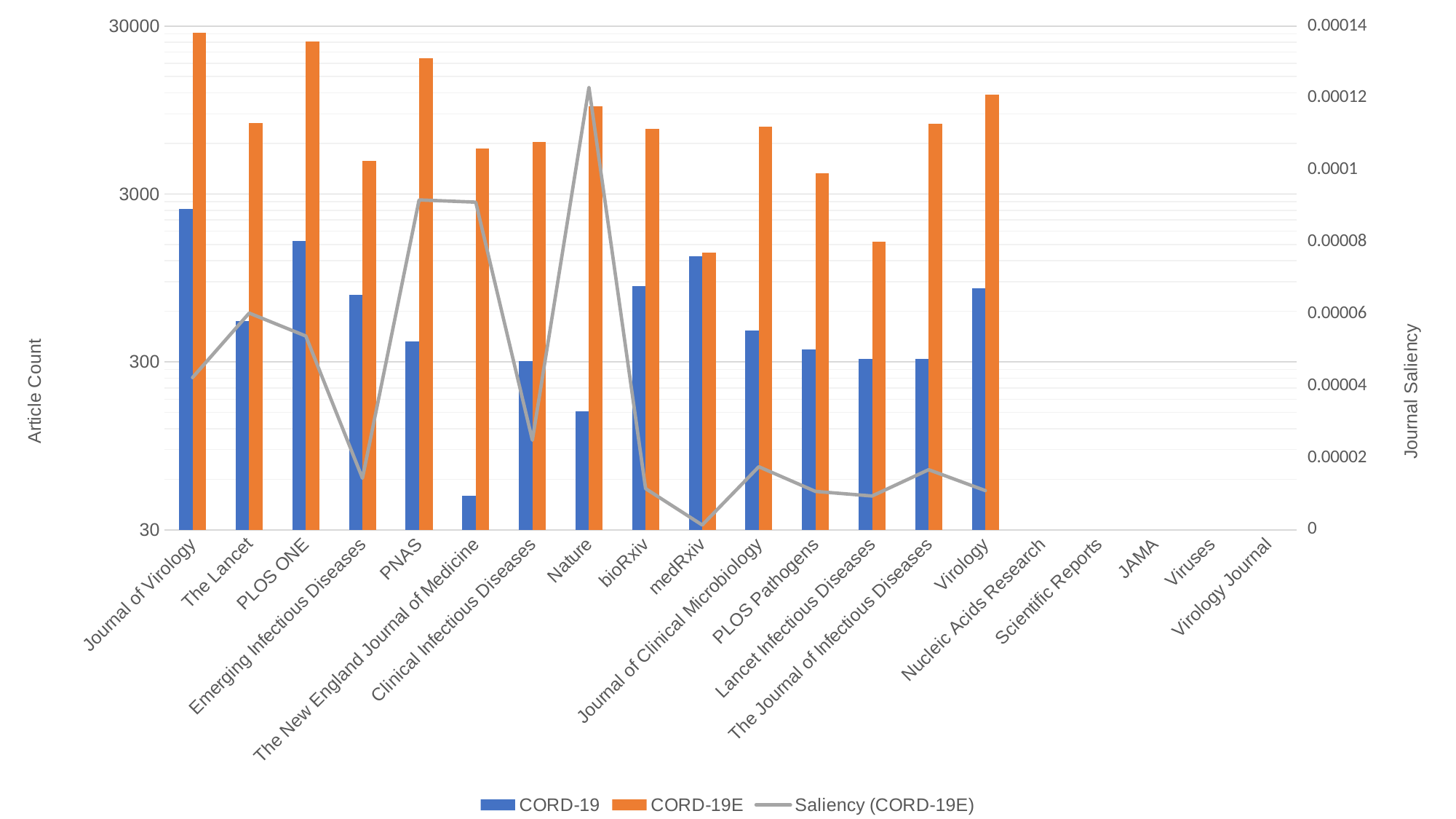

[unsupported chart]

## Slide 8
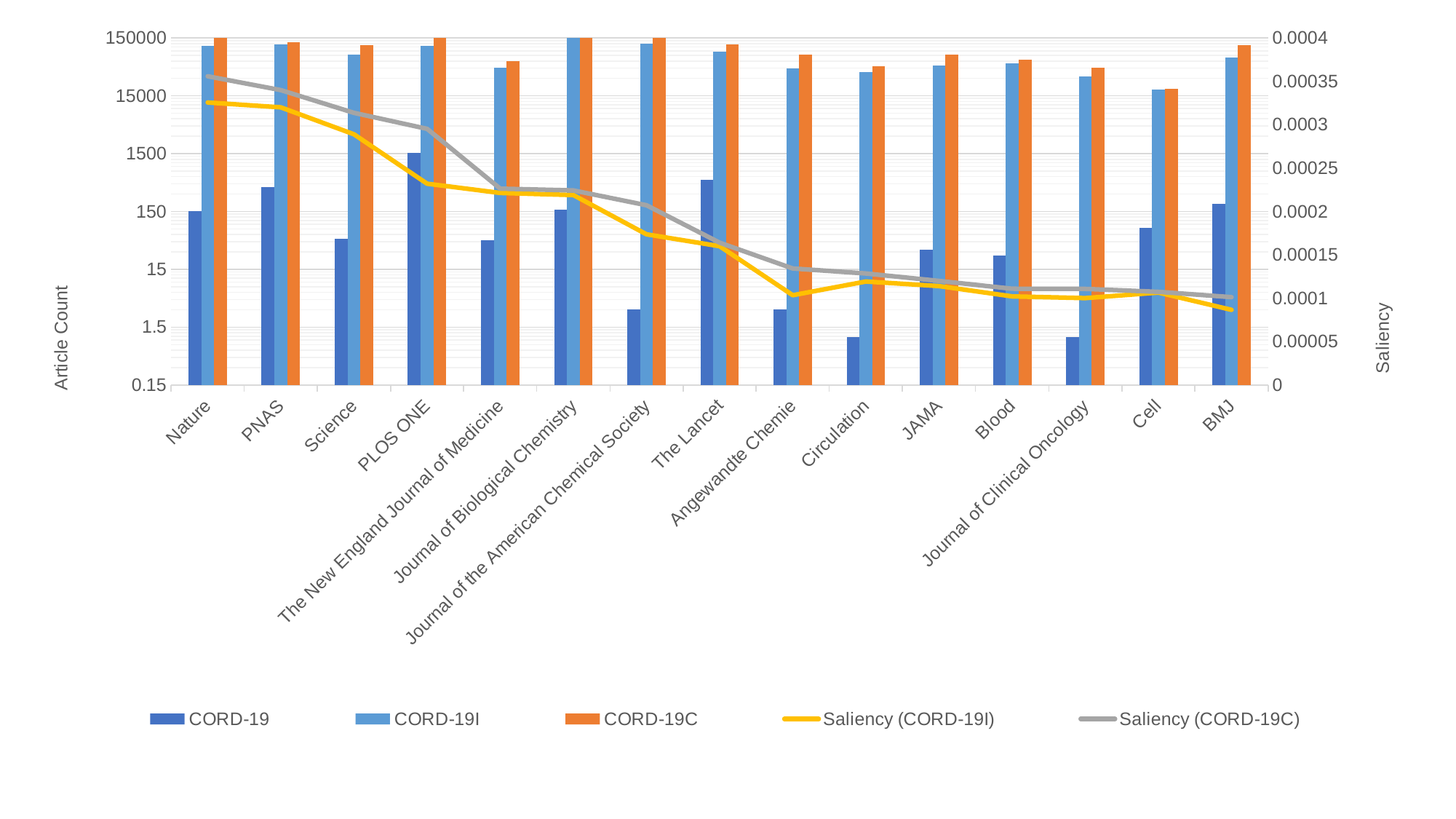

### Chart
| Category | | | | | |
|---|---|---|---|---|---|
| Nature | 152.0 | 108683.0 | 164708.0 | 0.00032565255234219445 | 0.0003558856947150451 |
| PNAS | 399.0 | 116146.0 | 126905.0 | 0.0003200525895966636 | 0.000339930757499906 |
| Science | 50.0 | 78202.0 | 111585.0 | 0.00028899509752822736 | 0.00031372100264455443 |
| PLOS ONE | 1567.0 | 109457.0 | 150267.0 | 0.00023203142170089082 | 0.00029524397161027183 |
| The New England Journal of Medicine | 48.0 | 46257.0 | 60213.0 | 0.00022138645096378792 | 0.00022633405301387177 |
| Journal of Biological Chemistry | 163.0 | 177125.0 | 185253.0 | 0.00021893619668393718 | 0.00022428475062656342 |
| Journal of the American Chemical Society | 3.0 | 118764.0 | 169779.0 | 0.00017373576881365448 | 0.00020722038097391505 |
| The Lancet | 524.0 | 86279.0 | 117769.0 | 0.00016000105876317998 | 0.00016406441865011 |
| Angewandte Chemie | 3.0 | 44472.0 | 77179.0 | 0.00010347585082903366 | 0.00013429944426894452 |
| Circulation | 1.0 | 38927.0 | 48802.0 | 0.00011927903966426084 | 0.000128567412232494 |
| JAMA | 33.0 | 50021.0 | 76981.0 | 0.00011406278095271708 | 0.0001199800671777121 |
| Blood | 26.0 | 53935.0 | 63923.0 | 0.00010215139981196968 | 0.00011096163123764918 |
| Journal of Clinical Oncology | 1.0 | 32629.0 | 45520.0 | 0.00010017949450604389 | 0.00011076374397793645 |
| Cell | 78.0 | 19063.0 | 19586.0 | 0.00010658024768723497 | 0.00010738494737542243 |
| BMJ | 204.0 | 68132.0 | 111476.0 | 8.665938853563127e-05 | 0.00010125803310190238 |

## Slide 9
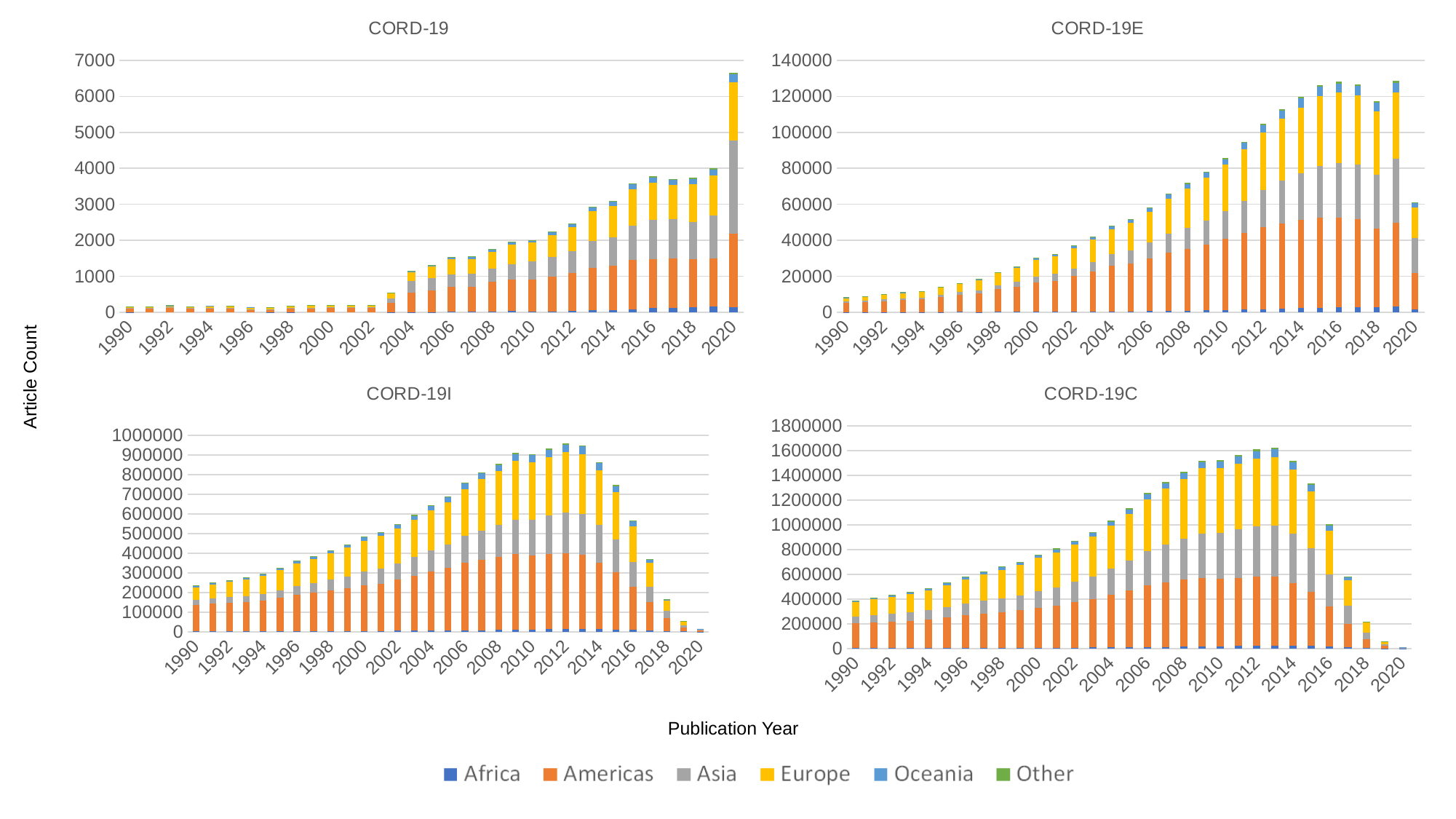

### Chart: CORD-19
| Category | Africa | Americas | Asia | Europe | Oceania | Other |
|---|---|---|---|---|---|---|
| 1990 | 2.0 | 107.0 | 2.0 | 41.0 | 1.0 | 2.0 |
| 1991 | None | 106.0 | 13.0 | 45.0 | None | 2.0 |
| 1992 | None | 115.0 | 13.0 | 56.0 | 2.0 | 5.0 |
| 1993 | None | 102.0 | 10.0 | 43.0 | 3.0 | 1.0 |
| 1994 | None | 113.0 | 12.0 | 47.0 | 1.0 | None |
| 1995 | None | 99.0 | 12.0 | 61.0 | 3.0 | 2.0 |
| 1996 | None | 81.0 | 6.0 | 51.0 | 2.0 | 1.0 |
| 1997 | 1.0 | 82.0 | 4.0 | 55.0 | 2.0 | 1.0 |
| 1998 | 2.0 | 97.0 | 13.0 | 64.0 | None | 2.0 |
| 1999 | None | 119.0 | 6.0 | 75.0 | 1.0 | 1.0 |
| 2000 | None | 127.0 | 16.0 | 61.0 | 1.0 | 2.0 |
| 2001 | None | 129.0 | 10.0 | 58.0 | 3.0 | 1.0 |
| 2002 | None | 119.0 | 14.0 | 63.0 | 8.0 | 3.0 |
| 2003 | 4.0 | 247.0 | 138.0 | 142.0 | 9.0 | 1.0 |
| 2004 | 3.0 | 541.0 | 319.0 | 238.0 | 22.0 | 17.0 |
| 2005 | 5.0 | 589.0 | 360.0 | 317.0 | 35.0 | 10.0 |
| 2006 | 11.0 | 693.0 | 339.0 | 437.0 | 46.0 | 16.0 |
| 2007 | 15.0 | 693.0 | 364.0 | 400.0 | 60.0 | 12.0 |
| 2008 | 16.0 | 823.0 | 378.0 | 461.0 | 67.0 | 17.0 |
| 2009 | 27.0 | 882.0 | 420.0 | 547.0 | 69.0 | 23.0 |
| 2010 | 22.0 | 877.0 | 513.0 | 528.0 | 55.0 | 14.0 |
| 2011 | 26.0 | 955.0 | 553.0 | 617.0 | 64.0 | 25.0 |
| 2012 | 38.0 | 1049.0 | 603.0 | 676.0 | 74.0 | 18.0 |
| 2013 | 50.0 | 1174.0 | 752.0 | 840.0 | 98.0 | 22.0 |
| 2014 | 63.0 | 1220.0 | 790.0 | 887.0 | 102.0 | 32.0 |
| 2015 | 76.0 | 1368.0 | 963.0 | 1011.0 | 141.0 | 27.0 |
| 2016 | 115.0 | 1369.0 | 1077.0 | 1040.0 | 131.0 | 42.0 |
| 2017 | 113.0 | 1390.0 | 1082.0 | 954.0 | 136.0 | 26.0 |
| 2018 | 133.0 | 1346.0 | 1036.0 | 1051.0 | 135.0 | 33.0 |
| 2019 | 156.0 | 1348.0 | 1187.0 | 1116.0 | 150.0 | 44.0 |
| 2020 | 132.0 | 2047.0 | 2603.0 | 1602.0 | 226.0 | 37.0 |
### Chart: CORD-19E
| Category | Africa | Americas | Asia | Europe | Oceania | Other |
|---|---|---|---|---|---|---|
| 1990 | 66.0 | 5228.0 | 630.0 | 2052.0 | 208.0 | 82.0 |
| 1991 | 55.0 | 5611.0 | 633.0 | 2393.0 | 215.0 | 99.0 |
| 1992 | 66.0 | 6136.0 | 860.0 | 2799.0 | 255.0 | 92.0 |
| 1993 | 76.0 | 6708.0 | 901.0 | 3101.0 | 236.0 | 82.0 |
| 1994 | 96.0 | 6950.0 | 935.0 | 3379.0 | 284.0 | 88.0 |
| 1995 | 93.0 | 8277.0 | 1146.0 | 4060.0 | 339.0 | 95.0 |
| 1996 | 146.0 | 9552.0 | 1444.0 | 4593.0 | 428.0 | 116.0 |
| 1997 | 138.0 | 10427.0 | 1717.0 | 5426.0 | 525.0 | 134.0 |
| 1998 | 152.0 | 12571.0 | 2288.0 | 6618.0 | 563.0 | 141.0 |
| 1999 | 181.0 | 13978.0 | 2642.0 | 7727.0 | 685.0 | 191.0 |
| 2000 | 232.0 | 16390.0 | 3191.0 | 9254.0 | 875.0 | 224.0 |
| 2001 | 249.0 | 17218.0 | 3754.0 | 9931.0 | 982.0 | 240.0 |
| 2002 | 288.0 | 19731.0 | 4194.0 | 11418.0 | 1065.0 | 283.0 |
| 2003 | 404.0 | 22134.0 | 5238.0 | 12580.0 | 1287.0 | 325.0 |
| 2004 | 427.0 | 25222.0 | 6608.0 | 13952.0 | 1366.0 | 414.0 |
| 2005 | 485.0 | 26362.0 | 7385.0 | 15399.0 | 1573.0 | 408.0 |
| 2006 | 619.0 | 29451.0 | 8591.0 | 17123.0 | 1924.0 | 468.0 |
| 2007 | 708.0 | 32535.0 | 10314.0 | 19657.0 | 2176.0 | 445.0 |
| 2008 | 871.0 | 34464.0 | 11465.0 | 22108.0 | 2426.0 | 507.0 |
| 2009 | 1038.0 | 36648.0 | 13315.0 | 23824.0 | 2810.0 | 584.0 |
| 2010 | 1116.0 | 39816.0 | 15052.0 | 26033.0 | 3104.0 | 587.0 |
| 2011 | 1402.0 | 42500.0 | 17942.0 | 28704.0 | 3603.0 | 666.0 |
| 2012 | 1734.0 | 45535.0 | 20581.0 | 32089.0 | 3978.0 | 772.0 |
| 2013 | 1851.0 | 47538.0 | 23784.0 | 34288.0 | 4507.0 | 774.0 |
| 2014 | 2174.0 | 49060.0 | 26160.0 | 36290.0 | 5153.0 | 810.0 |
| 2015 | 2502.0 | 50209.0 | 28618.0 | 38653.0 | 5418.0 | 788.0 |
| 2016 | 2930.0 | 49525.0 | 30410.0 | 39101.0 | 5250.0 | 865.0 |
| 2017 | 2866.0 | 48661.0 | 30447.0 | 38565.0 | 5434.0 | 811.0 |
| 2018 | 2894.0 | 43566.0 | 30048.0 | 35280.0 | 4787.0 | 880.0 |
| 2019 | 3209.0 | 46490.0 | 35631.0 | 36825.0 | 5389.0 | 944.0 |
| 2020 | 1486.0 | 20463.0 | 19412.0 | 16829.0 | 2432.0 | 425.0 |Article Count
### Chart: CORD-19I
| Category | Africa | Americas | Asia | Europe | Oceania | Other |
|---|---|---|---|---|---|---|
| 1990 | 2134.0 | 134440.0 | 24584.0 | 65838.0 | 6492.0 | 2304.0 |
| 1991 | 2105.0 | 139985.0 | 26773.0 | 71250.0 | 6914.0 | 2584.0 |
| 1992 | 2177.0 | 144498.0 | 29470.0 | 77669.0 | 7496.0 | 2598.0 |
| 1993 | 2282.0 | 149145.0 | 30895.0 | 83459.0 | 8051.0 | 2632.0 |
| 1994 | 2296.0 | 156085.0 | 34113.0 | 91564.0 | 8830.0 | 2607.0 |
| 1995 | 2598.0 | 170946.0 | 38256.0 | 101111.0 | 9988.0 | 2798.0 |
| 1996 | 2843.0 | 185731.0 | 44731.0 | 114216.0 | 11470.0 | 2848.0 |
| 1997 | 2996.0 | 194419.0 | 49624.0 | 123823.0 | 12315.0 | 2906.0 |
| 1998 | 3147.0 | 206034.0 | 55311.0 | 135172.0 | 13181.0 | 3000.0 |
| 1999 | 3461.0 | 217604.0 | 61202.0 | 145393.0 | 14843.0 | 3071.0 |
| 2000 | 3930.0 | 233053.0 | 68261.0 | 158851.0 | 16017.0 | 3383.0 |
| 2001 | 4217.0 | 240290.0 | 75559.0 | 167459.0 | 17262.0 | 3384.0 |
| 2002 | 4712.0 | 259665.0 | 83475.0 | 178556.0 | 18305.0 | 3830.0 |
| 2003 | 5382.0 | 280375.0 | 93574.0 | 191062.0 | 20141.0 | 4246.0 |
| 2004 | 6143.0 | 301586.0 | 107015.0 | 202439.0 | 22053.0 | 4481.0 |
| 2005 | 6722.0 | 318525.0 | 117757.0 | 217745.0 | 24254.0 | 4827.0 |
| 2006 | 7598.0 | 345358.0 | 134935.0 | 239054.0 | 27173.0 | 5331.0 |
| 2007 | 8259.0 | 359299.0 | 148787.0 | 260534.0 | 29149.0 | 5450.0 |
| 2008 | 9389.0 | 372584.0 | 160798.0 | 274964.0 | 31374.0 | 5814.0 |
| 2009 | 10620.0 | 385928.0 | 175709.0 | 300102.0 | 33528.0 | 6142.0 |
| 2010 | 10926.0 | 378437.0 | 180766.0 | 293916.0 | 34823.0 | 5834.0 |
| 2011 | 12263.0 | 382808.0 | 195913.0 | 298803.0 | 36864.0 | 6046.0 |
| 2012 | 13031.0 | 387731.0 | 206165.0 | 306966.0 | 37953.0 | 6110.0 |
| 2013 | 13100.0 | 378404.0 | 208816.0 | 303592.0 | 39615.0 | 5942.0 |
| 2014 | 12862.0 | 339159.0 | 193342.0 | 276059.0 | 37220.0 | 5353.0 |
| 2015 | 11598.0 | 290535.0 | 167774.0 | 240081.0 | 32515.0 | 4537.0 |
| 2016 | 9709.0 | 219101.0 | 125168.0 | 184064.0 | 24774.0 | 3571.0 |
| 2017 | 6333.0 | 143506.0 | 78713.0 | 121519.0 | 16443.0 | 2314.0 |
| 2018 | 3015.0 | 66018.0 | 35668.0 | 54145.0 | 7393.0 | 1157.0 |
| 2019 | 994.0 | 21898.0 | 10974.0 | 17379.0 | 2415.0 | 371.0 |
| 2020 | 213.0 | 4785.0 | 4911.0 | 3486.0 | 472.0 | 76.0 |
### Chart: CORD-19C
| Category | Africa | Americas | Asia | Europe | Oceania | Other |
|---|---|---|---|---|---|---|
| 1990 | 3929.0 | 199388.0 | 54991.0 | 116720.0 | 9816.0 | 4368.0 |
| 1991 | 4084.0 | 208007.0 | 60629.0 | 125240.0 | 10433.0 | 4769.0 |
| 1992 | 4098.0 | 214271.0 | 63970.0 | 135661.0 | 11309.0 | 4796.0 |
| 1993 | 4175.0 | 221109.0 | 68743.0 | 145795.0 | 12413.0 | 4776.0 |
| 1994 | 4398.0 | 231760.0 | 75333.0 | 159556.0 | 13725.0 | 4741.0 |
| 1995 | 4698.0 | 250020.0 | 82182.0 | 175160.0 | 14981.0 | 5026.0 |
| 1996 | 5067.0 | 262357.0 | 94856.0 | 195476.0 | 17081.0 | 5098.0 |
| 1997 | 5261.0 | 278930.0 | 104871.0 | 212686.0 | 18458.0 | 5247.0 |
| 1998 | 5378.0 | 289634.0 | 113435.0 | 227813.0 | 19675.0 | 5444.0 |
| 1999 | 5914.0 | 303516.0 | 122159.0 | 242527.0 | 21265.0 | 5335.0 |
| 2000 | 6669.0 | 323898.0 | 135577.0 | 265675.0 | 22953.0 | 5936.0 |
| 2001 | 7165.0 | 339321.0 | 147737.0 | 282981.0 | 24874.0 | 6191.0 |
| 2002 | 8039.0 | 366285.0 | 163718.0 | 301322.0 | 26556.0 | 6930.0 |
| 2003 | 8793.0 | 391249.0 | 182357.0 | 322873.0 | 29200.0 | 7635.0 |
| 2004 | 10443.0 | 424014.0 | 209847.0 | 347739.0 | 32263.0 | 8460.0 |
| 2005 | 11476.0 | 460596.0 | 238817.0 | 377732.0 | 35885.0 | 9116.0 |
| 2006 | 13161.0 | 495342.0 | 279247.0 | 418460.0 | 40295.0 | 9937.0 |
| 2007 | 14427.0 | 518152.0 | 306289.0 | 452125.0 | 43432.0 | 10569.0 |
| 2008 | 16225.0 | 539565.0 | 332100.0 | 479423.0 | 47280.0 | 11138.0 |
| 2009 | 18488.0 | 552671.0 | 360463.0 | 524158.0 | 50684.0 | 11811.0 |
| 2010 | 19760.0 | 546264.0 | 370170.0 | 522451.0 | 53191.0 | 11814.0 |
| 2011 | 22028.0 | 550537.0 | 391508.0 | 529315.0 | 55799.0 | 12355.0 |
| 2012 | 24084.0 | 559520.0 | 405147.0 | 547871.0 | 58972.0 | 12986.0 |
| 2013 | 25200.0 | 555540.0 | 413097.0 | 552879.0 | 62156.0 | 13107.0 |
| 2014 | 25190.0 | 506239.0 | 396182.0 | 518913.0 | 59628.0 | 12231.0 |
| 2015 | 22463.0 | 435586.0 | 352551.0 | 458701.0 | 53959.0 | 10562.0 |
| 2016 | 18069.0 | 322754.0 | 261575.0 | 351682.0 | 40625.0 | 8410.0 |
| 2017 | 10600.0 | 190167.0 | 146160.0 | 206997.0 | 23933.0 | 5070.0 |
| 2018 | 3883.0 | 73387.0 | 51809.0 | 79915.0 | 8795.0 | 2079.0 |
| 2019 | 1157.0 | 20807.0 | 12633.0 | 22081.0 | 2419.0 | 615.0 |
| 2020 | 192.0 | 3601.0 | 3594.0 | 3208.0 | 413.0 | 77.0 |Publication Year

## Slide 10
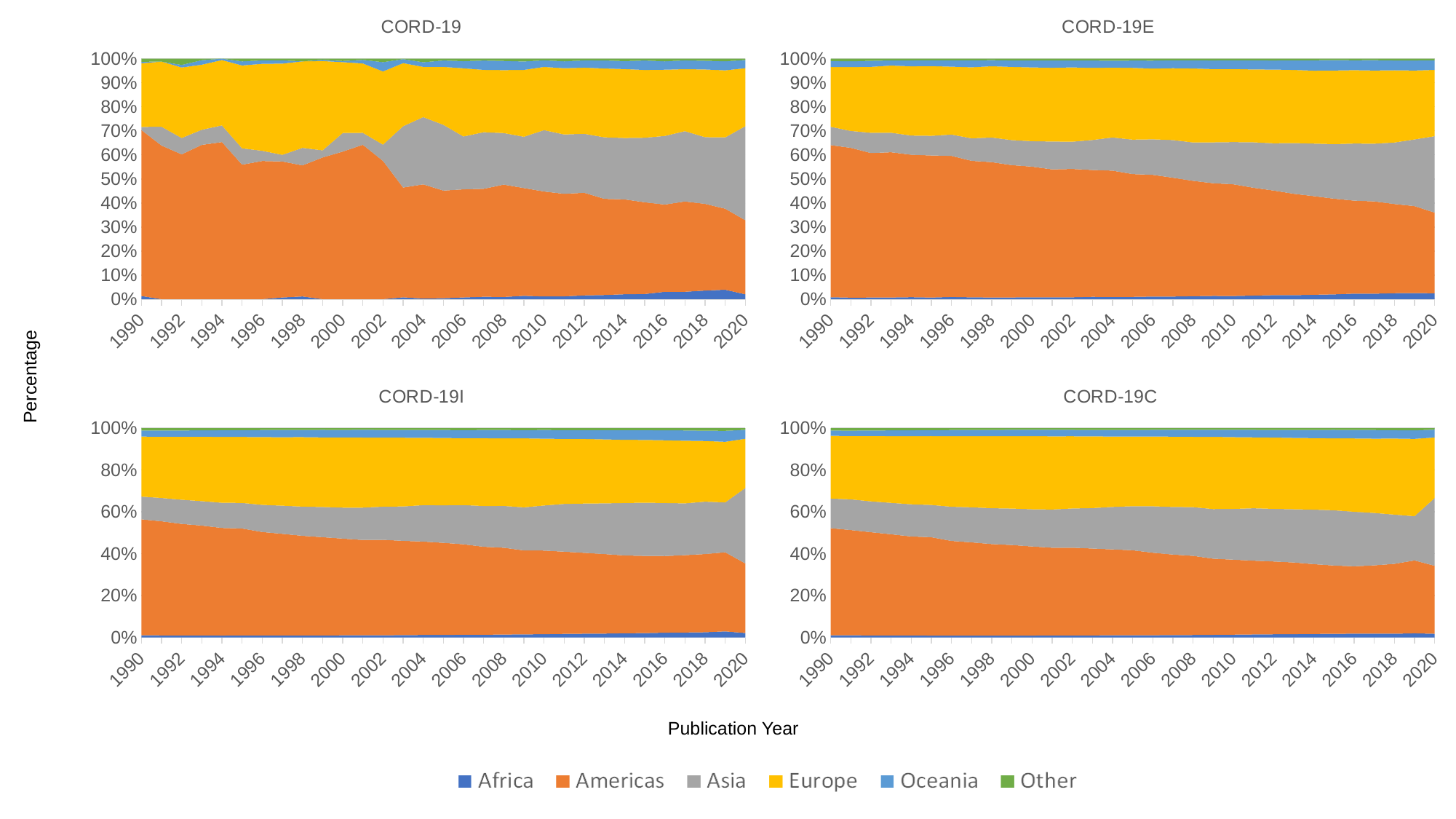

### Chart: CORD-19
| Category | Africa | Americas | Asia | Europe | Oceania | Other |
|---|---|---|---|---|---|---|
| 1990 | 0.012903225806451613 | 0.6903225806451613 | 0.012903225806451613 | 0.2645161290322581 | 0.0064516129032258064 | 0.012903225806451613 |
| 1991 | 0.0 | 0.6385542168674698 | 0.0783132530120482 | 0.2710843373493976 | 0.0 | 0.012048192771084338 |
| 1992 | 0.0 | 0.6020942408376964 | 0.06806282722513089 | 0.2931937172774869 | 0.010471204188481676 | 0.02617801047120419 |
| 1993 | 0.0 | 0.6415094339622641 | 0.06289308176100629 | 0.27044025157232704 | 0.018867924528301886 | 0.006289308176100629 |
| 1994 | 0.0 | 0.653179190751445 | 0.06936416184971098 | 0.27167630057803466 | 0.005780346820809248 | 0.0 |
| 1995 | 0.0 | 0.559322033898305 | 0.06779661016949153 | 0.3446327683615819 | 0.01694915254237288 | 0.011299435028248588 |
| 1996 | 0.0 | 0.574468085106383 | 0.0425531914893617 | 0.3617021276595745 | 0.014184397163120567 | 0.0070921985815602835 |
| 1997 | 0.006896551724137931 | 0.5655172413793104 | 0.027586206896551724 | 0.3793103448275862 | 0.013793103448275862 | 0.006896551724137931 |
| 1998 | 0.011235955056179775 | 0.5449438202247191 | 0.07303370786516854 | 0.3595505617977528 | 0.0 | 0.011235955056179775 |
| 1999 | 0.0 | 0.5891089108910891 | 0.0297029702970297 | 0.3712871287128713 | 0.0049504950495049506 | 0.0049504950495049506 |
| 2000 | 0.0 | 0.6135265700483091 | 0.07729468599033816 | 0.2946859903381642 | 0.004830917874396135 | 0.00966183574879227 |
| 2001 | 0.0 | 0.6417910447761194 | 0.04975124378109453 | 0.2885572139303483 | 0.014925373134328358 | 0.004975124378109453 |
| 2002 | 0.0 | 0.5748792270531401 | 0.06763285024154589 | 0.30434782608695654 | 0.03864734299516908 | 0.014492753623188406 |
| 2003 | 0.0073937153419593345 | 0.4565619223659889 | 0.25508317929759705 | 0.26247689463955637 | 0.0166358595194085 | 0.0018484288354898336 |
| 2004 | 0.002631578947368421 | 0.4745614035087719 | 0.27982456140350875 | 0.20877192982456141 | 0.01929824561403509 | 0.014912280701754385 |
| 2005 | 0.003799392097264438 | 0.44756838905775076 | 0.2735562310030395 | 0.24088145896656535 | 0.026595744680851064 | 0.007598784194528876 |
| 2006 | 0.007133592736705577 | 0.4494163424124514 | 0.2198443579766537 | 0.2833981841763943 | 0.029831387808041506 | 0.010376134889753566 |
| 2007 | 0.009715025906735751 | 0.4488341968911917 | 0.23575129533678757 | 0.25906735751295334 | 0.038860103626943004 | 0.007772020725388601 |
| 2008 | 0.009080590238365494 | 0.46708286038592506 | 0.21452894438138478 | 0.2616345062429058 | 0.038024971623155504 | 0.009648127128263337 |
| 2009 | 0.013719512195121951 | 0.4481707317073171 | 0.21341463414634146 | 0.2779471544715447 | 0.0350609756097561 | 0.011686991869918699 |
| 2010 | 0.010950721752115481 | 0.43653558984569435 | 0.25535092085614736 | 0.2628173220507715 | 0.0273768043802887 | 0.006968641114982578 |
| 2011 | 0.011607142857142858 | 0.4263392857142857 | 0.246875 | 0.2754464285714286 | 0.02857142857142857 | 0.011160714285714286 |
| 2012 | 0.015459723352318959 | 0.4267697314890155 | 0.2453213995117982 | 0.27502034174125306 | 0.030105777054515868 | 0.007323026851098454 |
| 2013 | 0.0170299727520436 | 0.39986376021798364 | 0.2561307901907357 | 0.28610354223433243 | 0.03337874659400545 | 0.007493188010899182 |
| 2014 | 0.020361990950226245 | 0.394311570782159 | 0.2553329023917259 | 0.28668390433096314 | 0.03296703296703297 | 0.010342598577892695 |
| 2015 | 0.021193530395984383 | 0.3814835471277189 | 0.26854433909648634 | 0.28192972671500277 | 0.03931957612939208 | 0.007529280535415505 |
| 2016 | 0.03047164811870694 | 0.3627450980392157 | 0.28537360890302066 | 0.27556968733439324 | 0.034711181770005296 | 0.011128775834658187 |
| 2017 | 0.03053228857065658 | 0.37557416914347475 | 0.2923534179951364 | 0.2577681707646582 | 0.03674682518238314 | 0.007025128343690895 |
| 2018 | 0.0356186395286556 | 0.3604713444027852 | 0.2774504552758436 | 0.2814675950723085 | 0.03615425816818425 | 0.008837707552222818 |
| 2019 | 0.03899025243689078 | 0.3369157710572357 | 0.29667583104223944 | 0.2789302674331417 | 0.03749062734316421 | 0.010997250687328168 |
| 2020 | 0.019858582819316985 | 0.3079584775086505 | 0.39160523544456144 | 0.2410109823980743 | 0.034000300887618476 | 0.0055664209417782455 |
### Chart: CORD-19E
| Category | Africa | Americas | Asia | Europe | Oceania | Other |
|---|---|---|---|---|---|---|
| 1990 | 66.0 | 5228.0 | 630.0 | 2052.0 | 208.0 | 82.0 |
| 1991 | 55.0 | 5611.0 | 633.0 | 2393.0 | 215.0 | 99.0 |
| 1992 | 66.0 | 6136.0 | 860.0 | 2799.0 | 255.0 | 92.0 |
| 1993 | 76.0 | 6708.0 | 901.0 | 3101.0 | 236.0 | 82.0 |
| 1994 | 96.0 | 6950.0 | 935.0 | 3379.0 | 284.0 | 88.0 |
| 1995 | 93.0 | 8277.0 | 1146.0 | 4060.0 | 339.0 | 95.0 |
| 1996 | 146.0 | 9552.0 | 1444.0 | 4593.0 | 428.0 | 116.0 |
| 1997 | 138.0 | 10427.0 | 1717.0 | 5426.0 | 525.0 | 134.0 |
| 1998 | 152.0 | 12571.0 | 2288.0 | 6618.0 | 563.0 | 141.0 |
| 1999 | 181.0 | 13978.0 | 2642.0 | 7727.0 | 685.0 | 191.0 |
| 2000 | 232.0 | 16390.0 | 3191.0 | 9254.0 | 875.0 | 224.0 |
| 2001 | 249.0 | 17218.0 | 3754.0 | 9931.0 | 982.0 | 240.0 |
| 2002 | 288.0 | 19731.0 | 4194.0 | 11418.0 | 1065.0 | 283.0 |
| 2003 | 404.0 | 22134.0 | 5238.0 | 12580.0 | 1287.0 | 325.0 |
| 2004 | 427.0 | 25222.0 | 6608.0 | 13952.0 | 1366.0 | 414.0 |
| 2005 | 485.0 | 26362.0 | 7385.0 | 15399.0 | 1573.0 | 408.0 |
| 2006 | 619.0 | 29451.0 | 8591.0 | 17123.0 | 1924.0 | 468.0 |
| 2007 | 708.0 | 32535.0 | 10314.0 | 19657.0 | 2176.0 | 445.0 |
| 2008 | 871.0 | 34464.0 | 11465.0 | 22108.0 | 2426.0 | 507.0 |
| 2009 | 1038.0 | 36648.0 | 13315.0 | 23824.0 | 2810.0 | 584.0 |
| 2010 | 1116.0 | 39816.0 | 15052.0 | 26033.0 | 3104.0 | 587.0 |
| 2011 | 1402.0 | 42500.0 | 17942.0 | 28704.0 | 3603.0 | 666.0 |
| 2012 | 1734.0 | 45535.0 | 20581.0 | 32089.0 | 3978.0 | 772.0 |
| 2013 | 1851.0 | 47538.0 | 23784.0 | 34288.0 | 4507.0 | 774.0 |
| 2014 | 2174.0 | 49060.0 | 26160.0 | 36290.0 | 5153.0 | 810.0 |
| 2015 | 2502.0 | 50209.0 | 28618.0 | 38653.0 | 5418.0 | 788.0 |
| 2016 | 2930.0 | 49525.0 | 30410.0 | 39101.0 | 5250.0 | 865.0 |
| 2017 | 2866.0 | 48661.0 | 30447.0 | 38565.0 | 5434.0 | 811.0 |
| 2018 | 2894.0 | 43566.0 | 30048.0 | 35280.0 | 4787.0 | 880.0 |
| 2019 | 3209.0 | 46490.0 | 35631.0 | 36825.0 | 5389.0 | 944.0 |
| 2020 | 1486.0 | 20463.0 | 19412.0 | 16829.0 | 2432.0 | 425.0 |Percentage
### Chart: CORD-19I
| Category | Africa | Americas | Asia | Europe | Oceania | Other |
|---|---|---|---|---|---|---|
| 1990 | 0.0102977773963786 | 0.554202149408635 | 0.108570880510446 | 0.28705559285415 | 0.0293436176495827 | 0.0105299821808067 |
| 1991 | 0.00958757159550217 | 0.545672662490661 | 0.111325019634886 | 0.292832787387698 | 0.0294571193226442 | 0.0111248395686071 |
| 1992 | 0.00926380479645084 | 0.533359697449044 | 0.115574828633247 | 0.300900925471372 | 0.0301596392661684 | 0.010741104383716 |
| 1993 | 0.00924417092831758 | 0.525413885758862 | 0.116659443529513 | 0.307987345063708 | 0.030545202392303 | 0.0101499523272947 |
| 1994 | 0.00880438995532527 | 0.514530507333591 | 0.12034434222068 | 0.315109850880235 | 0.0316435813222904 | 0.00956732828787662 |
| 1995 | 0.00903750564844103 | 0.511534023221161 | 0.122361104368252 | 0.315547123869844 | 0.0321167556101623 | 0.00940348728213823 |
| 1996 | 0.00920352513682551 | 0.494808755449683 | 0.129724067114511 | 0.323713771059719 | 0.033761240781781 | 0.0087886404574795 |
| 1997 | 0.00909363413028887 | 0.485966935793925 | 0.134648342554037 | 0.327455541871118 | 0.0344587153571963 | 0.00837683029343342 |
| 1998 | 0.00890095496946926 | 0.476568505070008 | 0.139755754106122 | 0.332219304772387 | 0.0344569956279247 | 0.00809848545408706 |
| 1999 | 0.00915940657203293 | 0.469874094231651 | 0.144293839765214 | 0.332889888003601 | 0.0358757852878114 | 0.00790698613968816 |
| 2000 | 0.00972253504510012 | 0.462394221178287 | 0.148514001952014 | 0.335561526003625 | 0.0357851496723403 | 0.00802256614863198 |
| 2001 | 0.00986720577972989 | 0.455766902015516 | 0.154947970116169 | 0.334912873034768 | 0.0368673289273839 | 0.00763772012643159 |
| 2002 | 0.0102368315162198 | 0.456178505048956 | 0.158611009982946 | 0.330531817049569 | 0.0362791967656019 | 0.00816263963670505 |
| 2003 | 0.0108916182091553 | 0.450625418908392 | 0.164635527054689 | 0.328632501856783 | 0.0366125935184682 | 0.00860234045251164 |
| 2004 | 0.0115262988884139 | 0.446452685819037 | 0.174268282049809 | 0.321930378602765 | 0.0370798057967501 | 0.00874254884322319 |
| 2005 | 0.0119319185850375 | 0.440113495799129 | 0.179535012610644 | 0.321673842150074 | 0.0379724975501614 | 0.00877323330495269 |
| 2006 | 0.0123935824909579 | 0.4325790596309 | 0.187591579337846 | 0.319929518686821 | 0.0386219048502272 | 0.00888435500324585 |
| 2007 | 0.0127207115232595 | 0.420389650441866 | 0.195036139794894 | 0.324056448268161 | 0.0390731622586396 | 0.0087238877131787 |
| 2008 | 0.0136115704308939 | 0.414932367881938 | 0.200040529885681 | 0.322721769146539 | 0.0398691463690842 | 0.00882461628586171 |
| 2009 | 0.0144813507077163 | 0.401254025070787 | 0.206124097647529 | 0.329650262024216 | 0.0395440438665934 | 0.00894622068315711 |
| 2010 | 0.0153054727035088 | 0.399772110325413 | 0.215573578203022 | 0.319261704480713 | 0.0413552733000435 | 0.0087318609872985 |
| 2011 | 0.0167968307111558 | 0.392804740594508 | 0.228532936727602 | 0.310774715050787 | 0.0420887827716604 | 0.00900199414428534 |
| 2012 | 0.0175759612217671 | 0.386646195201958 | 0.235620348591561 | 0.308781992208423 | 0.0421949491152135 | 0.00918055366107638 |
| 2013 | 0.0181745426438247 | 0.380100688471101 | 0.24257063412532 | 0.305824130347792 | 0.0440443658575791 | 0.00928563855438169 |
| 2014 | 0.0196581329035457 | 0.371885073146541 | 0.25089883461443 | 0.302159527646912 | 0.0459877882469625 | 0.00941064344160674 |
| 2015 | 0.020444177532424 | 0.369011514430572 | 0.254437733147008 | 0.300179010705995 | 0.0465081363253144 | 0.00941942785868548 |
| 2016 | 0.0225761204568658 | 0.366421439998982 | 0.253588547183073 | 0.299704519358911 | 0.0474772823414534 | 0.0102320906607125 |
| 2017 | 0.0233891064435742 | 0.369567207445455 | 0.247866551390937 | 0.299384631032904 | 0.0492193459797589 | 0.0105731577073691 |
| 2018 | 0.0244760593544439 | 0.37381296995799 | 0.250526588296207 | 0.290006001341476 | 0.0490697920711688 | 0.0121085889787128 |
| 2019 | 0.0286301625864324 | 0.378134928050831 | 0.238609605681181 | 0.289852364044103 | 0.0523266679125397 | 0.0124462717249112 |
| 2020 | 0.0209246961533642 | 0.332289186818694 | 0.361483523367999 | 0.235058263375516 | 0.0426011777972685 | 0.00764315248715699 |
### Chart: CORD-19C
| Category | Africa | Americas | Asia | Europe | Oceania | Other |
|---|---|---|---|---|---|---|
| 1990 | 0.01009475555738261 | 0.5122863632159337 | 0.14128803839552737 | 0.2998879787879099 | 0.025220188483397223 | 0.01122267555984913 |
| 1991 | 0.009884742546507182 | 0.5034514306736825 | 0.1467438922262938 | 0.30312565047124373 | 0.025251596226177626 | 0.011542687856095187 |
| 1992 | 0.009440112415199087 | 0.49359256401101115 | 0.1473606615910897 | 0.31250734269358793 | 0.026051300952534526 | 0.011048018336577555 |
| 1993 | 0.009135447505639908 | 0.4838154880298286 | 0.15041869889346207 | 0.3190185794215019 | 0.027161271829343275 | 0.01045051432022424 |
| 1994 | 0.00898443963694529 | 0.4734501433056936 | 0.15389376788767611 | 0.3259484426358442 | 0.028038070490467058 | 0.00968513604337372 |
| 1995 | 0.008829715054682963 | 0.4699032264733577 | 0.15445799119283848 | 0.32920666006348825 | 0.028156228444913892 | 0.009446178770718724 |
| 1996 | 0.008737186063955443 | 0.4523903540914068 | 0.16356315794011397 | 0.3370653607731901 | 0.02945330080095183 | 0.008790640330381853 |
| 1997 | 0.008411503342377445 | 0.44596476473851754 | 0.1676720712827343 | 0.3400511309402945 | 0.029511410129937823 | 0.008389119566138463 |
| 1998 | 0.008131494952213481 | 0.43792439735764216 | 0.171512854203112 | 0.3444515172087411 | 0.02974844982982526 | 0.008231286448466008 |
| 1999 | 0.008439938577112553 | 0.43315123388077337 | 0.17433453781560576 | 0.3461131185815651 | 0.030347530240496863 | 0.007613640904446309 |
| 2000 | 0.008766833002939367 | 0.42578492667357254 | 0.1782247590402625 | 0.34924701725234913 | 0.030173207064997345 | 0.007803256965879155 |
| 2001 | 0.008864623040101749 | 0.4198119685401766 | 0.1827819698639933 | 0.3501074518508071 | 0.030774408025051065 | 0.007659578679870192 |
| 2002 | 0.009210059002119493 | 0.4196425502663688 | 0.18756716503408374 | 0.34521624563212466 | 0.03042447155868706 | 0.007939508506616257 |
| 2003 | 0.009333334748600743 | 0.4152914690157275 | 0.19356293924150866 | 0.34271372572329895 | 0.030994356267387887 | 0.00810417500347625 |
| 2004 | 0.010111680671129763 | 0.4105615405619472 | 0.20318929941535643 | 0.3367064756198403 | 0.03123940950805894 | 0.008191594223667317 |
| 2005 | 0.010123303887892085 | 0.4063047470850072 | 0.21066722417172568 | 0.33320807112070866 | 0.031655172535465965 | 0.008041481199200439 |
| 2006 | 0.010474816983195405 | 0.3942418352777128 | 0.22225220105663454 | 0.33305158534974155 | 0.03207072033567805 | 0.007908840997037666 |
| 2007 | 0.01072644190234306 | 0.38524484124092745 | 0.22772517944317966 | 0.3361539159282495 | 0.03229159386584624 | 0.007858027619454064 |
| 2008 | 0.011380127106726303 | 0.3784479680949632 | 0.23293314096417908 | 0.3362646950932539 | 0.03316193587710445 | 0.00781213286377304 |
| 2009 | 0.0121769771615814 | 0.3640124483377517 | 0.23741614661375574 | 0.3452325830300835 | 0.03338262172531327 | 0.007779223131514383 |
| 2010 | 0.01296885767728809 | 0.35852328290617924 | 0.24294949627539134 | 0.3428943655038887 | 0.034910248416631116 | 0.007753749220621534 |
| 2011 | 0.014106569019597295 | 0.35255984149001435 | 0.25071884073563183 | 0.3389694289362694 | 0.03573326878175547 | 0.00791205103673164 |
| 2012 | 0.014972211515746807 | 0.3478347362269828 | 0.25186624227579607 | 0.3405929453306643 | 0.03666090589215333 | 0.008072958758656703 |
| 2013 | 0.015536575997593064 | 0.3425075170517004 | 0.2546870212253056 | 0.3408669286100498 | 0.038321088004222 | 0.008080869111129058 |
| 2014 | 0.016590017143237247 | 0.3334066569501898 | 0.260923627306154 | 0.3417536945553263 | 0.039270724184872985 | 0.00805527986021972 |
| 2015 | 0.016841077745006455 | 0.32656981216384195 | 0.2643163780474456 | 0.34389971075600795 | 0.04045442345380418 | 0.007918597833893879 |
| 2016 | 0.018012889848123097 | 0.3217517433195596 | 0.26076272411438367 | 0.35058991242280296 | 0.0404988460944159 | 0.008383884200714774 |
| 2017 | 0.01818409509252445 | 0.32622781240189597 | 0.2507346545965447 | 0.3550993520629513 | 0.04105659885371582 | 0.008697486992367826 |
| 2018 | 0.017660596357814688 | 0.3337775392508232 | 0.235636836647443 | 0.3634680808485091 | 0.04000127349136755 | 0.009455673404042426 |
| 2019 | 0.0193763397642015 | 0.3484559217577706 | 0.21156551446945338 | 0.3697916666666667 | 0.04051112004287245 | 0.01029943729903537 |
| 2020 | 0.017320703653585928 | 0.3248534055029319 | 0.32422192151556156 | 0.2894000902119982 | 0.0372575552548489 | 0.006946323861073523 |Publication Year

## Slide 11
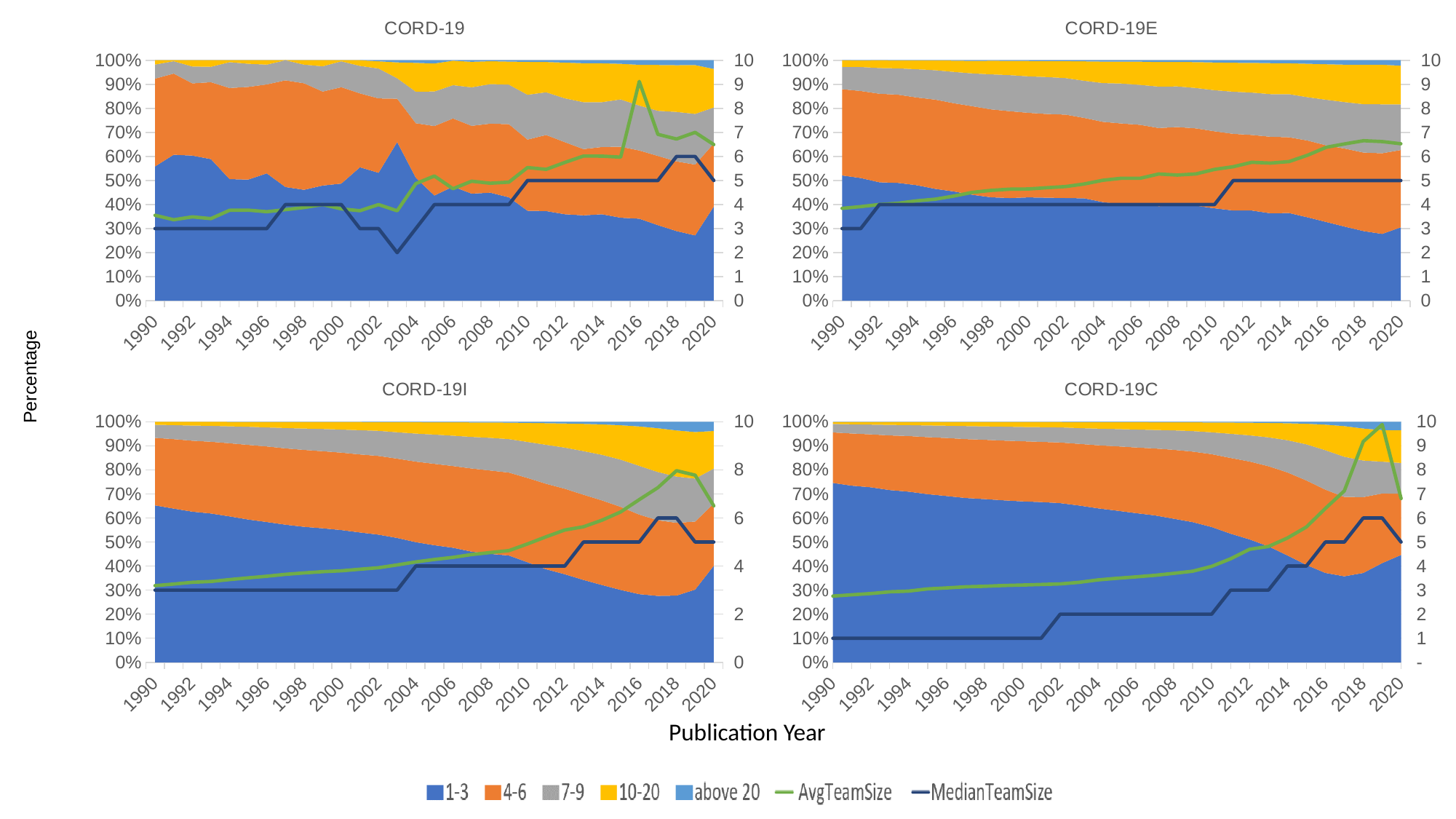

### Chart: CORD-19
| Category | 1-3 | 4-6 | 7-9 | 10-20 | above 20 | AvgTeamSize | MedianTeamSize |
|---|---|---|---|---|---|---|---|
| 1990 | 0.5585585585585585 | 0.36486486486486486 | 0.05855855855855856 | 0.018018018018018018 | 0.0 | 3.55405405405405 | 3.0 |
| 1991 | 0.6071428571428571 | 0.3373015873015873 | 0.051587301587301584 | 0.003968253968253968 | 0.0 | 3.36904761904761 | 3.0 |
| 1992 | 0.6037037037037037 | 0.3 | 0.07037037037037037 | 0.025925925925925925 | 0.0 | 3.48888888888888 | 3.0 |
| 1993 | 0.589041095890411 | 0.319634703196347 | 0.0639269406392694 | 0.0273972602739726 | 0.0 | 3.41552511415525 | 3.0 |
| 1994 | 0.5059760956175299 | 0.3784860557768924 | 0.10756972111553785 | 0.00796812749003984 | 0.0 | 3.7609561752988 | 3.0 |
| 1995 | 0.5037037037037037 | 0.3851851851851852 | 0.0962962962962963 | 0.014814814814814815 | 0.0 | 3.76666666666666 | 3.0 |
| 1996 | 0.5296803652968036 | 0.3698630136986301 | 0.0821917808219178 | 0.0182648401826484 | 0.0 | 3.70319634703196 | 3.0 |
| 1997 | 0.47280334728033474 | 0.4435146443514644 | 0.08368200836820083 | 0.0 | 0.0 | 3.78661087866108 | 4.0 |
| 1998 | 0.4611872146118721 | 0.4429223744292237 | 0.0776255707762557 | 0.0182648401826484 | 0.0 | 3.87671232876712 | 4.0 |
| 1999 | 0.4789915966386555 | 0.3907563025210084 | 0.10504201680672269 | 0.025210084033613446 | 0.0 | 4.0 | 4.0 |
| 2000 | 0.4870689655172414 | 0.40086206896551724 | 0.10775862068965517 | 0.004310344827586207 | 0.0 | 3.81896551724137 | 4.0 |
| 2001 | 0.5551181102362205 | 0.30708661417322836 | 0.1141732283464567 | 0.023622047244094488 | 0.0 | 3.74409448818897 | 3.0 |
| 2002 | 0.5321888412017167 | 0.3090128755364807 | 0.12446351931330472 | 0.030042918454935622 | 0.004291845493562232 | 4.0 | 3.0 |
| 2003 | 0.6599552572706935 | 0.18008948545861297 | 0.08501118568232663 | 0.06599552572706935 | 0.008948545861297539 | 3.74049217002237 | 2.0 |
| 2004 | 0.5113714679531358 | 0.22674017918676775 | 0.13094417643004824 | 0.11991729841488628 | 0.011026878015161957 | 4.8697450034459 | 3.0 |
| 2005 | 0.4375 | 0.28879310344827586 | 0.14367816091954022 | 0.11637931034482758 | 0.013649425287356323 | 5.1875 | 4.0 |
| 2006 | 0.4745356500898742 | 0.2834032354703415 | 0.1384062312762133 | 0.10185739964050329 | 0.0017974835230677051 | 4.65787896944278 | 4.0 |
| 2007 | 0.4453125 | 0.2819010416666667 | 0.16015625 | 0.10611979166666667 | 0.006510416666666667 | 4.97005208333333 | 4.0 |
| 2008 | 0.44925028835063435 | 0.28719723183391005 | 0.1643598615916955 | 0.09515570934256055 | 0.004036908881199538 | 4.88581314878892 | 4.0 |
| 2009 | 0.4301964839710445 | 0.30403309203722856 | 0.16442605997931747 | 0.09565667011375388 | 0.005687693898655636 | 4.93226473629782 | 4.0 |
| 2010 | 0.3742658836091831 | 0.29578216764548854 | 0.18633208756006406 | 0.1366791243993593 | 0.0069407367859049655 | 5.53977576081153 | 5.0 |
| 2011 | 0.37298091042584436 | 0.31620166421928536 | 0.1776798825256975 | 0.12579539892315222 | 0.007342143906020558 | 5.4645129711209 | 5.0 |
| 2012 | 0.3597122302158273 | 0.29991007194244607 | 0.1821043165467626 | 0.14838129496402877 | 0.009892086330935251 | 5.74955035971223 | 5.0 |
| 2013 | 0.3551779935275081 | 0.27548543689320387 | 0.19498381877022652 | 0.16181229773462782 | 0.012540453074433657 | 6.01699029126213 | 5.0 |
| 2014 | 0.35953800298062594 | 0.279806259314456 | 0.18628912071535023 | 0.1613263785394933 | 0.013040238450074515 | 6.01527570789865 | 5.0 |
| 2015 | 0.34532134532134534 | 0.29503829503829504 | 0.1968031968031968 | 0.14785214785214784 | 0.014985014985014986 | 5.97269397269397 | 5.0 |
| 2016 | 0.34119547657512117 | 0.2836833602584814 | 0.18675282714054928 | 0.16930533117932148 | 0.019063004846526656 | 9.11857835218093 | 5.0 |
| 2017 | 0.31430363864491845 | 0.28795483061480553 | 0.1875784190715182 | 0.19040150564617314 | 0.019761606022584692 | 6.91969887076537 | 5.0 |
| 2018 | 0.2897672935926044 | 0.2897672935926044 | 0.20561045584953777 | 0.19413452343002868 | 0.020720433535224736 | 6.72680905323557 | 6.0 |
| 2019 | 0.2716695753344968 | 0.29435718440954045 | 0.21087841768470042 | 0.20302501454333916 | 0.02006980802792321 | 7.00145433391506 | 6.0 |
| 2020 | 0.3919239904988123 | 0.26230064472344755 | 0.1493043773328809 | 0.16016287750254496 | 0.03630810994231422 | 6.49338310145911 | 5.0 |
### Chart: CORD-19E
| Category | | | | | | | |
|---|---|---|---|---|---|---|---|
| 1990 | 6359.0 | 4374.0 | 1134.0 | 326.0 | 10.0 | 3.842333852331394 | 3.0 |
| 1991 | 6801.0 | 4822.0 | 1328.0 | 361.0 | 14.0 | 3.9129521236680174 | 3.0 |
| 1992 | 7180.0 | 5367.0 | 1557.0 | 462.0 | 12.0 | 4.003155439703663 | 4.0 |
| 1993 | 7838.0 | 5870.0 | 1748.0 | 526.0 | 16.0 | 4.053381672709088 | 4.0 |
| 1994 | 8086.0 | 6135.0 | 1977.0 | 606.0 | 23.0 | 4.154573007666251 | 4.0 |
| 1995 | 8668.0 | 6915.0 | 2277.0 | 747.0 | 26.0 | 4.222884130306445 | 4.0 |
| 1996 | 8935.0 | 7212.0 | 2561.0 | 920.0 | 34.0 | 4.352761672261215 | 4.0 |
| 1997 | 9538.0 | 7972.0 | 2957.0 | 1121.0 | 63.0 | 4.507089741813311 | 4.0 |
| 1998 | 10314.0 | 8764.0 | 3489.0 | 1356.0 | 52.0 | 4.588237747653806 | 4.0 |
| 1999 | 11178.0 | 9463.0 | 3925.0 | 1554.0 | 66.0 | 4.638814633773772 | 4.0 |
| 2000 | 12649.0 | 10352.0 | 4467.0 | 1875.0 | 94.0 | 4.647484458334749 | 4.0 |
| 2001 | 13483.0 | 10959.0 | 4829.0 | 2080.0 | 121.0 | 4.699002287747839 | 4.0 |
| 2002 | 14961.0 | 12149.0 | 5320.0 | 2468.0 | 131.0 | 4.747552028319393 | 4.0 |
| 2003 | 16506.0 | 12957.0 | 5988.0 | 3134.0 | 175.0 | 4.85454076367389 | 4.0 |
| 2004 | 17477.0 | 14216.0 | 6864.0 | 3807.0 | 240.0 | 5.011266547741996 | 4.0 |
| 2005 | 17903.0 | 14986.0 | 7400.0 | 4076.0 | 252.0 | 5.0945603693659365 | 4.0 |
| 2006 | 19475.0 | 15769.0 | 7993.0 | 4652.0 | 288.0 | 5.092492268094734 | 4.0 |
| 2007 | 20035.0 | 16592.0 | 8778.0 | 5207.0 | 384.0 | 5.271687975527493 | 4.0 |
| 2008 | 21588.0 | 17591.0 | 9160.0 | 5503.0 | 407.0 | 5.226345186086379 | 4.0 |
| 2009 | 23321.0 | 18985.0 | 9918.0 | 6342.0 | 463.0 | 5.274949601043555 | 4.0 |
| 2010 | 23751.0 | 19786.0 | 10546.0 | 7130.0 | 575.0 | 5.46297015601735 | 4.0 |
| 2011 | 24942.0 | 21189.0 | 11638.0 | 7987.0 | 707.0 | 5.57355220197704 | 5.0 |
| 2012 | 26753.0 | 22416.0 | 12560.0 | 8804.0 | 789.0 | 5.756863239954011 | 5.0 |
| 2013 | 26898.0 | 23529.0 | 13107.0 | 9518.0 | 907.0 | 5.725996836084858 | 5.0 |
| 2014 | 28032.0 | 24206.0 | 13775.0 | 9855.0 | 981.0 | 5.788715533058335 | 5.0 |
| 2015 | 26471.0 | 24394.0 | 13766.0 | 10611.0 | 1125.0 | 6.054644021632381 | 5.0 |
| 2016 | 23767.0 | 23131.0 | 13764.0 | 10716.0 | 1212.0 | 6.3820636451301835 | 5.0 |
| 2017 | 22162.0 | 23359.0 | 13912.0 | 11194.0 | 1335.0 | 6.524109946916428 | 5.0 |
| 2018 | 18138.0 | 20435.0 | 12602.0 | 10257.0 | 1177.0 | 6.654282930569088 | 5.0 |
| 2019 | 18983.0 | 22898.0 | 13892.0 | 11234.0 | 1284.0 | 6.619334905038731 | 5.0 |
| 2020 | 9686.0 | 10195.0 | 6026.0 | 5092.0 | 719.0 | 6.528154360300145 | 5.0 |Percentage
### Chart: CORD-19I
| Category | 1-3 | 4-6 | 7-9 | 10-20 | above 20 | AvgTeamSize | MedianTeamSize |
|---|---|---|---|---|---|---|---|
| 1990 | 0.652414476945959 | 0.280473475458601 | 0.0544918195339613 | 0.012223599405057 | 0.000396628656420426 | 3.18694595934556 | 3.0 |
| 1991 | 0.638970815020433 | 0.28871973552317 | 0.0582470699360012 | 0.0136310625337342 | 0.000431316986660498 | 3.25334451384069 | 3.0 |
| 1992 | 0.626400242900452 | 0.294723815745285 | 0.0628067883315775 | 0.0155838121569836 | 0.000485340865700892 | 3.32570742456514 | 3.0 |
| 1993 | 0.618716392552502 | 0.298059744627187 | 0.0661420839682623 | 0.0165825933126653 | 0.000499185539383111 | 3.36007697966475 | 3.0 |
| 1994 | 0.606696712264624 | 0.30379151476638 | 0.0705207532024482 | 0.0183680892337536 | 0.000622930532793738 | 3.43764841633499 | 3.0 |
| 1995 | 0.593552049719134 | 0.310264531293573 | 0.0752798693279152 | 0.0202900282857256 | 0.000613521373650452 | 3.50985418907613 | 3.0 |
| 1996 | 0.583225964783115 | 0.314214062212405 | 0.0789350573654825 | 0.0227525461684765 | 0.000872369470519663 | 3.57865704951224 | 3.0 |
| 1997 | 0.572256582435059 | 0.317138047451195 | 0.0846271891072441 | 0.0249607053505826 | 0.00101747565591837 | 3.65628109857609 | 3.0 |
| 1998 | 0.563254121188503 | 0.319686607343956 | 0.0888285492629945 | 0.0271135056289839 | 0.00111721657556137 | 3.71500073289407 | 3.0 |
| 1999 | 0.557169358377874 | 0.320394935841284 | 0.092437856352316 | 0.0288508779751757 | 0.0011469714533492 | 3.76762113574651 | 3.0 |
| 2000 | 0.549914608754357 | 0.321654284100517 | 0.0959757495546621 | 0.0312037324830972 | 0.0012516251073657 | 3.80233179929882 | 3.0 |
| 2001 | 0.539920471513804 | 0.324241387703014 | 0.100741016043836 | 0.0337453378151702 | 0.00135178692417494 | 3.8696051861256 | 3.0 |
| 2002 | 0.530679904824391 | 0.327105286040647 | 0.104476573678094 | 0.03626000964404 | 0.00147822581282657 | 3.93091072938981 | 3.0 |
| 2003 | 0.517060214595629 | 0.3295196962986 | 0.109674888081375 | 0.0420700615954745 | 0.00167513942891998 | 4.0480840113842 | 3.0 |
| 2004 | 0.499402695558065 | 0.334900025429793 | 0.116496543322313 | 0.0470791221398875 | 0.00212161354993997 | 4.17260324200292 | 4.0 |
| 2005 | 0.486590440778643 | 0.338424812073351 | 0.121625666233063 | 0.0510711531156108 | 0.00228792779933002 | 4.27497150882363 | 4.0 |
| 2006 | 0.476000977945127 | 0.339812450014986 | 0.126223985347398 | 0.0555039110898701 | 0.00245867560261724 | 4.36233005095536 | 4.0 |
| 2007 | 0.45999014198283 | 0.345664526199049 | 0.13138272382491 | 0.0600052028423949 | 0.00295740515081397 | 4.47917220039158 | 4.0 |
| 2008 | 0.450542570951585 | 0.346706877053153 | 0.135755829608487 | 0.0638510420593462 | 0.00314368032742743 | 4.56250471215466 | 4.0 |
| 2009 | 0.443827721458826 | 0.344873060390977 | 0.139172659803936 | 0.0683126250202382 | 0.00381393332602144 | 4.64254657411904 | 4.0 |
| 2010 | 0.415792421443319 | 0.350345936396744 | 0.150016300667909 | 0.078910558095859 | 0.00493478339616753 | 4.92017966958406 | 4.0 |
| 2011 | 0.3866581038311 | 0.355451751973436 | 0.162063662570521 | 0.0898809746657182 | 0.00594550695922381 | 5.21988296178114 | 4.0 |
| 2012 | 0.36600577357167 | 0.35546285998639 | 0.171050304416512 | 0.10028508134261 | 0.00719598068281628 | 5.50066399071911 | 4.0 |
| 2013 | 0.342338535335818 | 0.354333869033537 | 0.181466948385149 | 0.113020778097747 | 0.0088398691477472 | 5.63357127124105 | 5.0 |
| 2014 | 0.321087122659533 | 0.351944975162399 | 0.189570118456247 | 0.126371799770729 | 0.011025983951089 | 5.90990638135269 | 5.0 |
| 2015 | 0.300857035112908 | 0.345282793480068 | 0.196415396182574 | 0.142894619112421 | 0.0145501561120258 | 6.2502594059545 | 5.0 |
| 2016 | 0.283281368024197 | 0.330735622468978 | 0.202614792310687 | 0.163862092832114 | 0.0195061243640211 | 6.76328442937432 | 5.0 |
| 2017 | 0.275874718137387 | 0.314682749468641 | 0.200508161878149 | 0.182091339670072 | 0.0268430308457496 | 7.25999331082028 | 6.0 |
| 2018 | 0.277292211052042 | 0.302560273919703 | 0.192207190392214 | 0.191743792397698 | 0.036196532238341 | 7.96594024740304 | 6.0 |
| 2019 | 0.301958307012002 | 0.283175405348494 | 0.178648136449778 | 0.19334596757212 | 0.0428721836176037 | 7.78092229943145 | 5.0 |
| 2020 | 0.401461693548387 | 0.258820564516129 | 0.14516129032258 | 0.155997983870967 | 0.0385584677419354 | 6.50214213709677 | 5.0 |
### Chart: CORD-19C
| Category | 1-3 | 4-6 | 7-9 | 10-20 | above 20 | AvgTeamSize | MedianTeamSize |
|---|---|---|---|---|---|---|---|
| 1990 | 0.7456450553947098 | 0.209986611270239 | 0.03520236152868579 | 0.008449638689821577 | 0.0007163331165437907 | 2.75289496495121 | 1.0 |
| 1991 | 0.7342866738407574 | 0.21679503716979096 | 0.03821262270166036 | 0.010023892344263569 | 0.0006817739435277364 | 2.80514174544214 | 1.0 |
| 1992 | 0.7275340752492675 | 0.2200367483855029 | 0.04049174126357316 | 0.011128739013120564 | 0.0008086960885358162 | 2.85691869419707 | 1.0 |
| 1993 | 0.7159893926663866 | 0.22719029436476024 | 0.04369148712716798 | 0.012281139350657144 | 0.0008476864910280261 | 2.9283967472844 | 1.0 |
| 1994 | 0.7094130620902022 | 0.23115943050216856 | 0.04542964249600992 | 0.0131188144974615 | 0.0008790504141578204 | 2.95930648383357 | 1.0 |
| 1995 | 0.6990743787706197 | 0.23679281103657765 | 0.04867653883474666 | 0.014404083871239927 | 0.0010521874868160148 | 3.05131924229 | 1.0 |
| 1996 | 0.6912784131476121 | 0.24130405151039164 | 0.050815072761024496 | 0.01548768060441629 | 0.0011147819765555023 | 3.09262020822245 | 1.0 |
| 1997 | 0.6831755009106761 | 0.24479378055149648 | 0.05406319867329193 | 0.016772427194411313 | 0.0011950926701241578 | 3.137954823945 | 1.0 |
| 1998 | 0.6786657823426864 | 0.24637669398086703 | 0.05574118325794693 | 0.01798059848273023 | 0.0012357419357694542 | 3.1589326435803 | 1.0 |
| 1999 | 0.6735706766552649 | 0.24815780539920634 | 0.0579374837747088 | 0.019043142870999306 | 0.001290891299820559 | 3.19223297093283 | 1.0 |
| 2000 | 0.6689347650117328 | 0.24996630043115328 | 0.05944134497829479 | 0.020379353196295125 | 0.0012782363825239737 | 3.21136302516838 | 1.0 |
| 2001 | 0.6662279707679971 | 0.2500338068836529 | 0.061103164641745436 | 0.021231357805073 | 0.0014036999015314994 | 3.23444772249532 | 1.0 |
| 2002 | 0.6618004057868417 | 0.25206925316012996 | 0.06266320114958844 | 0.022073644452686086 | 0.0013934954507537982 | 3.25721377017533 | 2.0 |
| 2003 | 0.6518791273320681 | 0.25618368697370786 | 0.06566309970895161 | 0.02475104334531631 | 0.0015230426399560953 | 3.3249400123729 | 2.0 |
| 2004 | 0.6400440111080933 | 0.26215876148085737 | 0.06905032454034998 | 0.026927148867295976 | 0.0018197540034033779 | 3.42618328353906 | 2.0 |
| 2005 | 0.6304719733255597 | 0.26742530794977476 | 0.07187374325212836 | 0.028301696090968862 | 0.0019272793815683362 | 3.49273666224787 | 2.0 |
| 2006 | 0.6202500249296024 | 0.2728786680050193 | 0.07457266612099943 | 0.030371595337632867 | 0.0019270456067460263 | 3.55177840443458 | 2.0 |
| 2007 | 0.6107665960142533 | 0.27816850328283693 | 0.07702796439356019 | 0.03187379409401277 | 0.0021631422153368714 | 3.61488938961166 | 2.0 |
| 2008 | 0.5967990107735716 | 0.28647603529476495 | 0.08081819682401171 | 0.03365763769099416 | 0.002249119416657577 | 3.69691233550297 | 2.0 |
| 2009 | 0.5827602383922701 | 0.29315816468783573 | 0.08521724318733091 | 0.03633027016585258 | 0.002534083566710725 | 3.78754045446024 | 2.0 |
| 2010 | 0.5623909674778524 | 0.30279951660218113 | 0.09099525373512528 | 0.04070433791066886 | 0.0031099242741723272 | 3.98800569161948 | 2.0 |
| 2011 | 0.534688558274989 | 0.314843196033566 | 0.10039611371816712 | 0.04650276110300206 | 0.0035693708702758805 | 4.3028313610931 | 3.0 |
| 2012 | 0.5108258227215878 | 0.32385150584098044 | 0.10882830362863359 | 0.05224540510223507 | 0.004248962706563145 | 4.69775101273 | 3.0 |
| 2013 | 0.48170957342835435 | 0.33341242079463307 | 0.119908381892309 | 0.059952830496705536 | 0.005016793387998006 | 4.8141593401929 | 3.0 |
| 2014 | 0.44415473286709234 | 0.34513071362094594 | 0.13405636605484875 | 0.0704698557484189 | 0.006188331708694099 | 5.16794820981979 | 4.0 |
| 2015 | 0.40465868701245167 | 0.35127588915591434 | 0.1502018245876012 | 0.08562106753151807 | 0.00824253171251468 | 5.6250278043507 | 4.0 |
| 2016 | 0.3715674622677759 | 0.3462171714012528 | 0.16408490979116588 | 0.10611489897861617 | 0.012015557561189202 | 6.39621516080316 | 5.0 |
| 2017 | 0.357424315233061 | 0.33007168028191575 | 0.16689091782796733 | 0.12749078968444658 | 0.018122296972609322 | 7.12414504244754 | 5.0 |
| 2018 | 0.37138267240325074 | 0.31532868625554483 | 0.15206950605343028 | 0.1330642919876819 | 0.028154843300092273 | 9.17521595570823 | 6.0 |
| 2019 | 0.4122129212277313 | 0.2895685769478429 | 0.13234599699506333 | 0.1308649924876583 | 0.035007512341704225 | 9.88323674608285 | 6.0 |
| 2020 | 0.4462564862861379 | 0.25436831515408237 | 0.1281372445197501 | 0.1363973313565604 | 0.034840622683469234 | 6.80790003176956 | 5.0 |Publication Year

## Slide 12
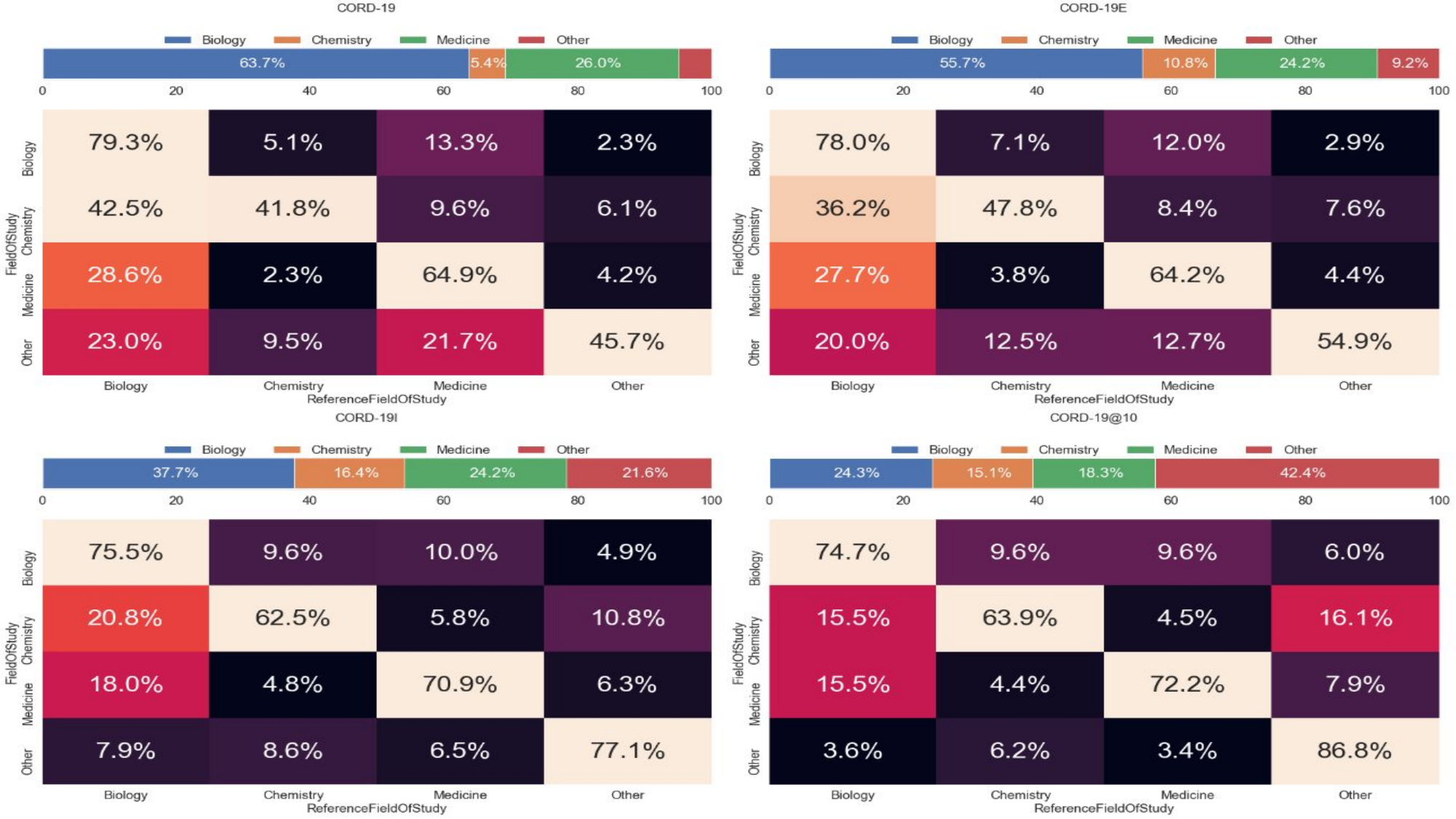

## Slide 13
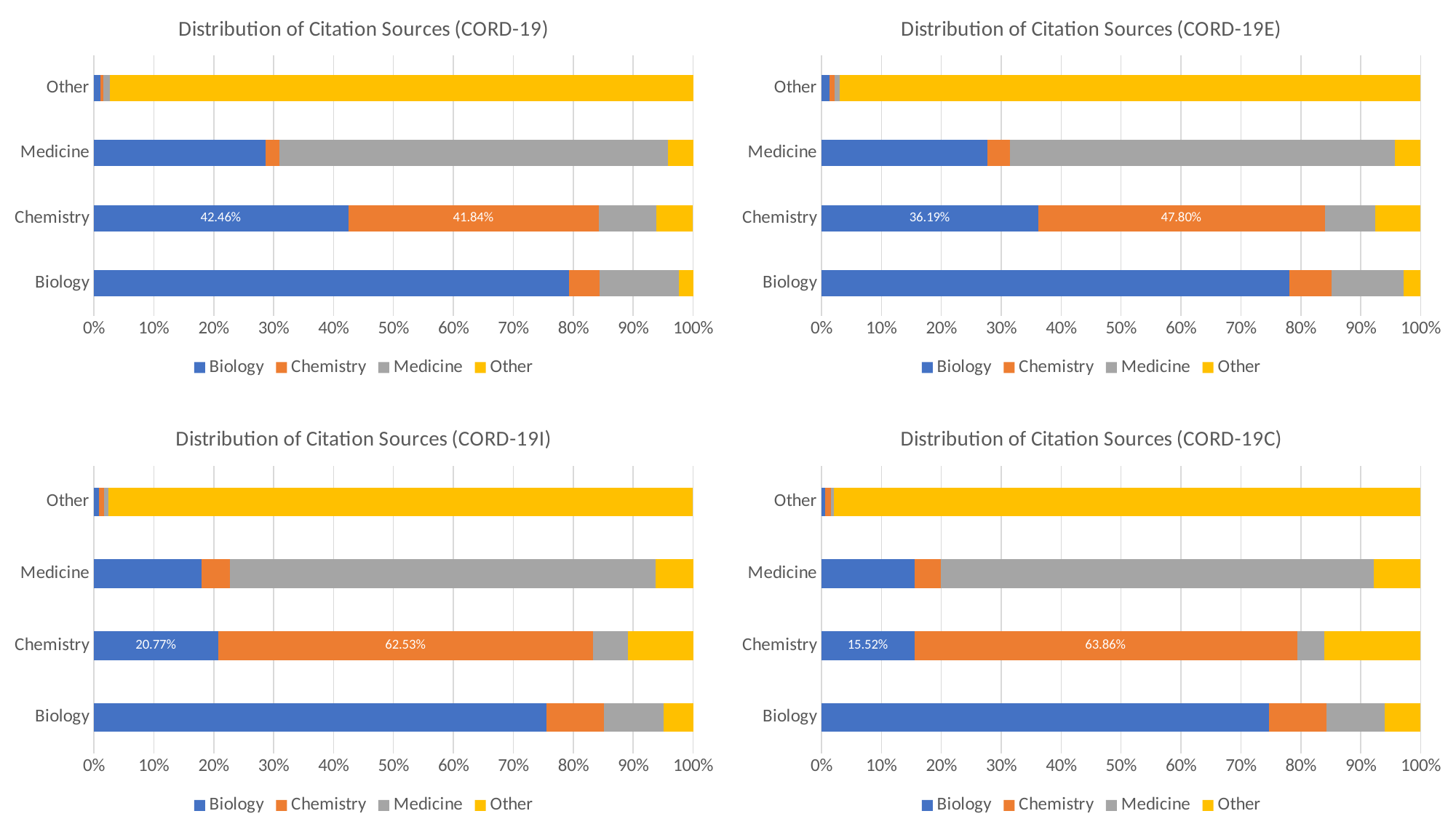

### Chart: Distribution of Citation Sources (CORD-19)
| Category | Biology | Chemistry | Medicine | Other |
|---|---|---|---|---|
| Biology | 0.7927425268247678 | 0.05070771183287166 | 0.13308243027972913 | 0.023467331062631457 |
| Chemistry | 0.4246465586312062 | 0.4183918740792432 | 0.09574147303851886 | 0.06122009425103168 |
| Medicine | 0.28646157477949225 | 0.023299067894213505 | 0.6487148671945977 | 0.0415244901316966 |
| Other | 0.011289685427365533 | 0.0046582001901460494 | 0.010647044703222842 | 0.9734050696792655 |
### Chart: Distribution of Citation Sources (CORD-19E)
| Category | Biology | Chemistry | Medicine | Other |
|---|---|---|---|---|
| Biology | 0.7804270766362582 | 0.07067500244084496 | 0.12022484789320771 | 0.02867307302968917 |
| Chemistry | 0.36190181853234477 | 0.47802507177922526 | 0.08363457829419857 | 0.0764385313942314 |
| Medicine | 0.276624093050244 | 0.03802288098843308 | 0.6416847614620623 | 0.043668264499260666 |
| Other | 0.013474795941611484 | 0.008434516240884708 | 0.008558881818442002 | 0.9695318059990619 |
### Chart: Distribution of Citation Sources (CORD-19I)
| Category | Biology | Chemistry | Medicine | Other |
|---|---|---|---|---|
| Biology | 0.7551242629875718 | 0.09610089573659597 | 0.09953958630679526 | 0.04923525496903687 |
| Chemistry | 0.2077124753328608 | 0.6253445703152546 | 0.05849663352679845 | 0.10844632082508614 |
| Medicine | 0.1795253238097599 | 0.04803476347658596 | 0.7093487096663451 | 0.06309120304730896 |
| Other | 0.00811798416725719 | 0.00887678215645592 | 0.00667143954415973 | 0.9763337941321272 |
### Chart: Distribution of Citation Sources (CORD-19C)
| Category | Biology | Chemistry | Medicine | Other |
|---|---|---|---|---|
| Biology | 0.7471783591327037 | 0.09601757308776622 | 0.09649805066916872 | 0.0603060171103614 |
| Chemistry | 0.15518327395881273 | 0.6385911399952708 | 0.045311741104760245 | 0.16091384494115624 |
| Medicine | 0.15537589139376232 | 0.04402244788259816 | 0.7218612551905595 | 0.07874040553307994 |
| Other | 0.005594958237735424 | 0.009645036909738992 | 0.005346684050253538 | 0.9794133208022721 |

## Slide 14
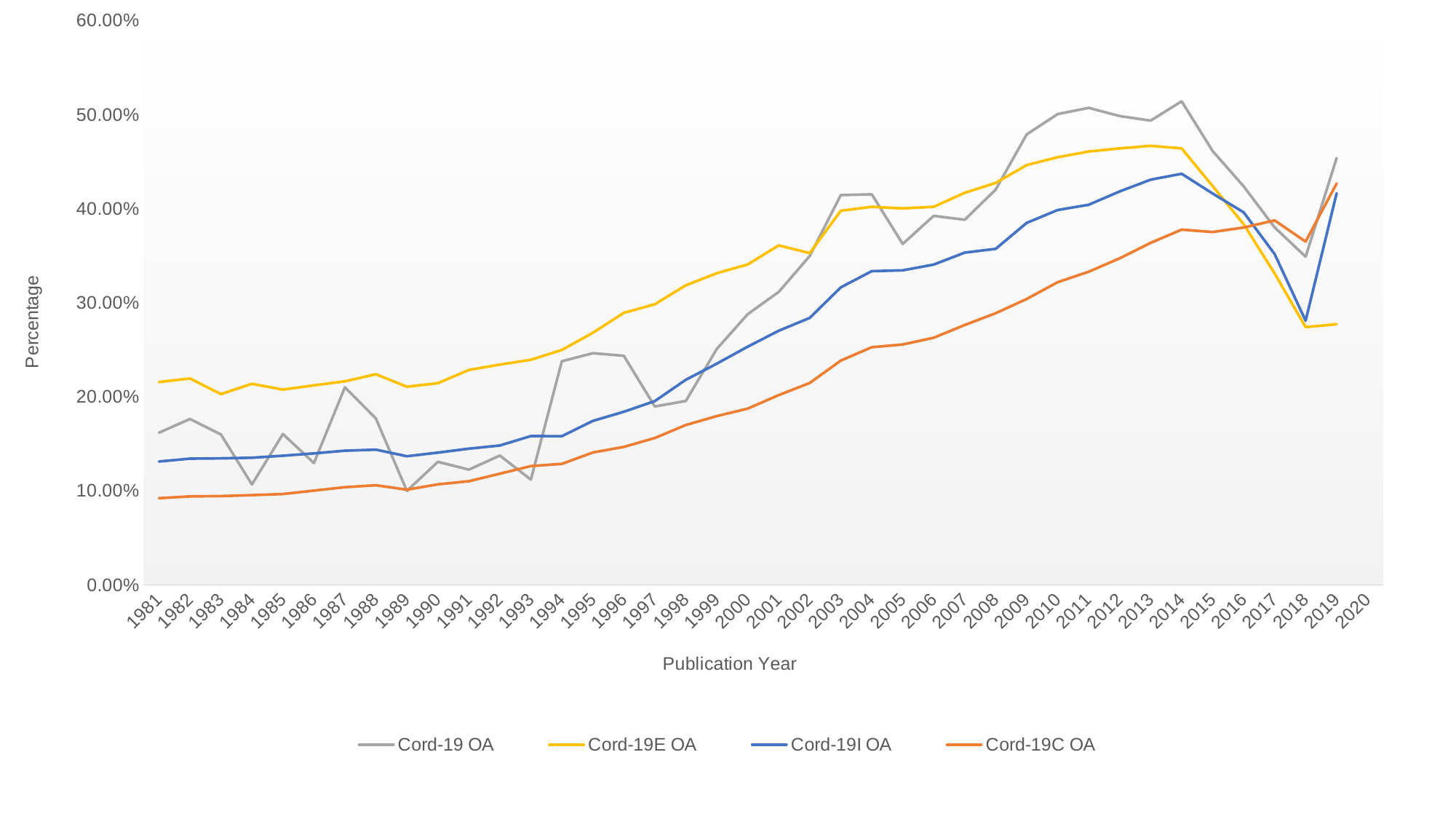

[unsupported chart]
