# Supplementary material for: Mitigating Biases in CORD-19 for Analyzing COVID-19 Literature
Source: Front Res Metr Anal. 2020 Nov 23;5:596624. doi: 10.3389/frma.2020.596624 (PMC8025972; doi:10.3389/frma.2020.596624)
Supplement: Supplementary file 1 [file DataSheet1.zip › CORD-19 Expansions Ver 1.2.docx]

Mitigating Biases in CORD-19 for Analyzing COVID-19 Literature

Anshul Kanakia^1^, Kuansan Wang^1,^*, Yuxiao Dong^1^, Boya Xie^1^, Kyle Lo^2^, Zhihong Shen^1^, Lucy Wang^2^, Chiyuan Huang^1^, Darrin Eide^1^, Sebastian Kohlmeier^2^, and Chieh-Han Wu^1^

^1^Microsoft Research, Redmond, Washington, USA

^2^Allen Institute for Artificial Intelligence, Seattle, Washington, USA

Author Note

*Corresponding Author: [kuansanw@microsoft.com](mailto:kuansanw@microsoft.com).

All Microsoft Research authors are employees of Microsoft Corporation that has commercial interests in the Azure platform used in conducting this research and distributing related data.

Abstract

On the behest of the Office of Science and Technology Policy in the White House, six institutions, including ours, have created an open research dataset called CORD-19 to facilitate the development of question-answering systems that can assist researchers in finding relevant research on COVID-19. As of May 27^th^, 2020, CORD-19 includes more than 100 thousand open access publications from major publishers and PubMed as well as preprint articles deposited into medRxiv and bioRxiv. Recent years, however, have also seen question-answering and other machine learning systems to exhibit horrifying behaviors that even cause harms on humans because of biases in their data. It is thus imperative and only ethical for modern scientists to be vigilant in inspecting and be prepared to mitigate the potential biases when working with any datasets. This article describes a framework to examine biases in scientific document collections like CORD-19 by comparing their properties with those derived from the citation behaviors from the entire scientific communities. In total, three expanded sets are created for the analyses: (1) the enclosure set CORD-19E composed of CORD-19 articles and their references and citations, mirroring the methodology used in the renowned “A Century of Physics” analysis, (2) the full closure graph CORD-19C that recursively includes references starting with CORD-19, and (3) the inflection closure CORD-19I that is a much smaller subset of CORD-19C but already appropriate for statistical analysis based on theory of the scale-free nature of the citation network. Taken together, all these expanded datasets show much smoother trends when used to analyze global COVID-19 research. The results suggest that, while CORD-19 exhibits a strong tilt towards recent and highly focused articles, the knowledge being explored to attack the pandemic encompasses a much longer time span and is very interdisciplinary. A question-answering system with such extended knowledge may perform better in understanding the literature and answering related questions. However, while CORD-19 appears to have topical coverage biases compared to the expanded sets, the collaboration patterns, especially in terms of team sizes and geographical distributions, are captured very well already in CORD-19 as the raw statistics and trends agree with those from larger datasets.

Keywords: COVID-19, CORD-19, Closure Graph, Citation Analysis, Scientometrics, Microsoft Academic Services

Mitigating Biases in CORD-19 for Analyzing COVID-19 Literature

Since first reported to the World Health Organization (WHO) in December 2019, the COVID-19 pandemic has been wreaking havoc around the globe, causing staggering loss of lives and livelihood. A key reason for its lasting power is that modern medicine has yet to find effective means to prevent or treat COVID-19 caused by a novel coronavirus. Nevertheless, the response from the research community has been swift and intense since the onset of the outbreak. Major publishers have all expedited the peer review and publication of research on COVID-19 [1], resulting in an impressive growth in literature on this subject that has surpassed 3,500 new titles per week by mid-March 2020 [2]. After 3 months, the growth in the research literature only sees acceleration and no sign of abating [3]. Facing the daunting task of tracking the voluminous new studies arriving at an unprecedented rate and motivated by the remarkable advancements in artificial intelligence in recent years, the United States Office of Science and Technology Policy in the White House (WH/OSTP) has challenged the research community to develop intelligent agents that can effectively sift through the literature and assist scientists and policy makers alike. Specifically, WH/OSTP has led the launch of an open question-answering challenge hosted on Kaggle [4] and three new tracks in the long running Text REtrieval Conference (TREC) organized by the National Institute of Standards and Technology that has given birth to pivotal theoretical and technological components behind modern web search engines and conversational systems. The COVID-19 Research Dataset (CORD-19) has been created to support these efforts. It comprises of research articles whose full-text contents are made publicly available for the purpose of text and data mining without infringing on the rights of the owners. The genesis and the details of CORD-19 are described in [5], and a comparison to other collections [6] indicates many relevant articles still are not included into CORD-19. The [6] study is based on running the same article retrieval query used for CORD-19 on other commercial search engines from Web of Science and Dimensions. As noted in [7], the keyword search approach can inadvertently include biases from the search engine designers and their content curators. Because the biases are implicit and undisclosed, it is difficult to tease apart whether the differences in corpus coverage are merely the result of search engines having different design approaches or whether substantive contents are indeed missing from CORD-19.

An approach to mitigate the search and the curation biases is to crowd-source to the domain experts by exploiting their citation behaviors in their scholarly communications. This is the method employed by [8] to analyze Physics literature in “A Century of Physics”. Their approach starts with a seed collection of research articles from a few hand-picked journals that is then expanded to include articles citing and cited by the seed collection. In other words, the corpus is constructed by a single-step traversal on the citation network in either direction, effectively forming an “enclosure” of the seed collection. The enclosure provides a more holistic view into how pivotal research is inspired by the prior art and how the impact is felt throughout the research community. The larger size also lends itself to more robust analytics using statistical methods. The motivation behind creating the CORD-19 corpus shares the same objectives, namely, to understand what knowledge has been exploited to attack the COVID-19 pandemic, where the potentially impactful research activities are taking place, and what opportunities exist for broader collaborations. These studies, however, must be conducted with extreme caution, especially given recent years have seen ample instances in which biases in datasets or methodologies have led to unintended and sometimes harmful consequences to the societies [9]. While the rest of this article is devoted to the data biases of CORD-19, the enclosure corpus using CORD-19 as the seed collection, called CORD-19E below, appears to be a reasonable starting point but with important drawbacks:

1. By design, the enclosure is susceptible to the selection bias in the seed collection: just consider the extreme case where the seed collection consists of a single article that is unlikely to cite all the relevant prior art.
2. Following both citations and references over multiple expansion steps from a seed collection quickly results in an explosion of included literature that quickly loses topical focus. Furthermore, there does not seem a straightforward enhancement to generalize the single-step traversal as described in [8] to a multi-step algorithm that can systemically augment the article collection without immediate topical digression from the seeds.

The latter problem is particularly noticeable when CORD-19 articles utilize advanced techniques from other fields like optics, instrumentation, big data analytics, or machine learning and cite the pertinent literature. More than one-hop traversal of the citation and reference networks together – a bi-directional graph – quickly grows the collection to include articles that bear little relevance to COVID-19 research as these techniques are widely adopted in diverse fields of study. For example, if an article in the original CORD-19 dataset utilizes the ImageNet technique for medical image analysis and references [10, 11] and this citation is subsequently bi-directionally expanded, the resulting dataset would include all 80,000 plus articles that also cite ImageNet that have little to do with the problem of COVID-19. Simply put, the bi-directional citation enclosure for more than one hops generalizes the resultant collection too quickly and loses the focus of the initial seed literature in just two iterations.

To overcome this difficulty, we posit that even though articles sometimes reference work outside of their main theme, their references overall are dominated by relevant work. This observation motivates another method to follow only the references but not the citations, iteratively, thereby augment the seed collection with unidirectional multi-step traversals on the citation network. The iterative traversal will eventually converge to an article collection, known as the “Closure Graph” [12] in the network science literature, where all references are made to articles within the collection itself.

The motivation of using a closure graph is further bolstered by a widely observed phenomenon that citation networks assume a power-law distribution [13], i.e., appear to be scale free. The mechanisms towards forming scale-free networks have seen many theoretical developments, ranging from the preferential attachment [14], homophily [15], node fitness [16], to temporal stochastic models [17], just to name a few. Despite remnant controversies [18, 19], these theories have recently been unified into a single mathematical framework, called Discrete Choice model [20], in which a new member is modeled to connect to an existing network by employing a logit utility function that evaluates the plausible choices. In the context of citation networks, this mechanism is consistent with the notion that a scholarly article will primarily reference the prior art that is most important and relevant to its contents. One can thus trace the COVID-19 research by following the references recursively from articles in a seed collection, turning the quest into a problem of solving the closure of CORD-19. Any initial selection bias in the seed collection may thus be alleviated by following the iterative expansion to a closure, provided the assumption about citing predominantly relevant work holds. Due to these desirable theoretical and mathematical properties, the closure graph of CORD-19 – henceforth called CORD-19C – is discussed and primarily analyzed in this work.

The closure graph method is a variant of the community detection approaches based on network traversing, with a difference that the manner in which the underlying network is explored here is systematic and deterministic as opposed to random walks, e.g., [21, 22, 23]. Network traversing, while being straightforward from a computational point of view, does have an unsolved problem in over-generalization, namely, how to avoid including the entire network by drawing a clear boundary within which a community shares strong common properties among its constituents. This issue is particularly critical here because, modern research being highly interdisciplinary in nature, a full closure inevitably will contain articles that are rather remote to the core research topics of the seeds, and this can take place as early as the first hop as noted previously in CORD-19E.

To address this issue, this work further proposes a pragmatic yet theoretically well-motivated approach by using the rate of encountering new articles during network traversal as a stop criterion to avoid over-generalization. If scholarly articles indeed make predominantly relevant references as hypothesized in the Discrete Choice model, the acceleration of reaching new articles by walking the citation networks should decrease from the initial quasi-exponential expansions to an inflection point beyond which the newly encountered articles are less relevant to the seeds. The empirical evidence below suggests a partial closure terminated at the inflection point, which is called the “Inflection Closure” or CORD-19I, leads to a collection that maintains topical focus to the initial seed while retaining many desirable properties of the full closure. Namely, the inflection closure already provides a large enough landscape from which broader trends, lineages, and other aggregate properties of the research represented by the seeds can be derived.

The main contribution of this work is to provide a critical inspection on the CORD-19 dataset and demonstrate the areas in which CORD-19 will be a biased source for literature analyses. Most interestingly, the method of using the full closure to identify biases in CORD-19 leads to a discovery that a partial closure at the infection point seems to be an economic yet effective means to mitigate these biases. In the following, the citation data and the methods of computing the closure graphs are first described in detail before demonstrating their effectiveness. As there are potentially unlimited areas for which CORD-19 and its expanded datasets can be used, this article is scoped to mainly demonstrate a few rudimentary areas where the biases can emerge, especially for the purposes of identifying important publication venues to follow and vital trending topics to track. To balance the unintended perception that CORD-19 is a totally biased dataset, an area that it seems to sample the research articles well, namely, in describing the patterns of collaborations, is also provided. The raw data behind these analyses are embedded into the figures included in the supplementary materials.

# Data and Methods

As the research activities on COVID-19 are ongoing, CORD-19 is a fast-growing dataset. Since its first release on March 16, 2020, CORD-19 has more than doubled its size from 29,000 articles to more than 60,000 on April 17^th^, 2020, and then again to almost 120,000 articles in May, 2020. Although the analyses reported in this article are solely based on the April 17^th^, 2020 release of CORD-19, the most up-to-date versions of CORD-19 and the corresponding closure graphs are released weekly at [<https://aka.ms/magcord19mapping>](https://aka.ms/magcord19mapping).

For this work, the snapshot of Microsoft Academic Graph [7, 24], or MAG, taken on the same date is used to obtain the enclosure, the inflection, and the closure graphs. The articles in CORD-19 are first identified in MAG for expanding their citation network. Once CORD-19 articles are mapped to MAG, the citation network in MAG is traversed for creating the enclosure CORD-19E as well as the full/inflection closures CORD-19C/CORD-19I using a breadth-first search algorithm. The articles in the April 17^th^ version of CORD-19 are mapped to 48,526 unique seed articles in MAG. The discrepancies can be largely attributed to the following:

1. MAG is updated approximately on a weekly basis based on the web crawl from the week before [25]. Therefore, the April 17^th^, 2020 version of MAG only contains content published before April 10^th^, 2020. Articles published after April 10^th^ are not available in the April 17^th^ version of MAG.
2. Unlike CORD-19 in which articles with distinct DOIs are treated as unique articles, MAG combines articles of the same contents into a single entity even though they are assigned distinct DOIs. The motivations behind this design choice are described in [5]. Consequently, multiple articles in CORD-19 may be mapped to the same article in MAG.
3. Similarly, articles with the same contents but significantly different publication dates are treated as a single entity in MAG but as distinct in CORD-19. A major source of the discrepancies can be observed between the records in PubMed and from the publishers themselves.
4. CORD-19 honors unique identification assignments on articles tracked by the World Health Organization (WHO). Unfortunately, WHO’s data systematically treats Chinese journals in English translation and transliteration as they are distinct (e.g., “Chinese Journal of Stomatology” vs “Zhonghua Kou Qiang Yi Xue Za Zhi”), leading to duplicate entries for every article in those journals from MAG’s perspective.
5. MAG fails to recognize some articles as distinct, mostly because they have same titles and author lists (e.g., “Infectious disease surveillance update” by R. Heald).

### Construction of CORD-19E

All referenced articles are admitted into the collection for the purpose of computing the enclosure regardless of the properties – such as citation count, venue, importance, etc. – of the citing or the cited articles. In total, the CORD-19E contains 926,281 references and 505,060 citations, i.e., articles cited by and citing CORD-19, respectively. In total, these 1,479,867 articles correspond to 0.6% of articles indexed by MAG. Within CORD-19E, the citation edges terminated at the seed articles, the references, and the citations are 1,362,025, 136,692,815, and 7,903,302, accounting for 0.085%, 8.5%, and 0.5% of the citation edges in MAG, respectively. Please note that the terms “references” and “citations” are not interchangeable in this article: when article A cites article B, A is called a citation of B while B, a reference of A.

### Construction and Convergence of CORD-19I and CORD-19C

To construct the closure graphs, literature abounds with iterative algorithmic designs, e.g., beam search, best-first, or the mathematically optimal A* search [12], but those algorithms require additional heuristics that can accurately assess the importance of each article, itself an actively researched topic. This study reports only the results for CORD-19C and CORD-19I with the most straightforward breadth-first algorithm as no material differences have been observed in the converged outcomes from these other alternatives for this application.

Each iterative citation expansion step of the current collection of articles in the graph is called a “hop”. Figure 1 shows the cumulative number of articles at the end of each hop, with hop 0 being the CORD-19 collection. Not surprisingly, the collection exhibits an exponential growth pattern in the early hops because each article typically cites more than one other article. However, as elaborated earlier, the growth eventually slows when most important articles are encountered after a few hops. Based on the recent CORD-19 releases, the inflection point leading to CORD-19I typically takes place after 3 hops, although it takes approximately 11 hops to see the growth rate reaching below 0.5% of the total article counts, at which point the closure is considered reached in this work. As shown in Figure 1 the inflection and the closure graphs, denoted for the rest of the article as CORD-19I and CORD-19C respectively, have more than 22 million and 59 million nodes (articles), and 731 million and 966 million edges (citation links). In other words, they cover approximately 9% and 25% of the articles but 45% and 60% of the citation links of MAG, respectively. In addition to different search algorithms, variations in the seed document collection, such as the experiments mentioned in [6], are also replicated with MAG and no substantial differences are observed in the converged closure, suggesting the closure graphs are relatively stable and reliable bases to broadly understand the COVID-19 research.

Figure 1*: Accumulated paper and citation counts per hop of the closure graph expansion. The callouts are article counts for CORD-19 (seed), CORD-19I (hop 3), and CORD-19C (hop 11).*

It is long known that citation networks must be analyzed carefully because not all nodes and edges are equally important [26]. Particularly, since it takes time for a publication to receive its due recognition, using the simple citation count as a measure for article importance has an intrinsic age bias favoring older articles. To mitigate this bias, MAG uses a measure, called saliency, that utilizes reinforcement learning to acquire the optimal strategy in assessing article importance [25]. Figure 2 shows the aggregate saliencies of articles from the seed collection to the closure graphs. As saliency is a probabilistic measure, the information theoretical entropy that quantifies the information amount can be computed from it and shown also in Figure 2. As can be seen, the full closure CORD-19C eventually accounts for 68.1% of all the probability mass and its information amount reaches 11.68 nits, out of 16.0 nits of the entire MAG, or 73%. In contrast, the partial closure at the inflection point, CORD-19I, amounts to 38.6% and 6.46/16 = 40.37% of the saliencies and information of the entire MAG. These measures show CORD-19I and CORD-19C account for more important contents out of the entire scholarly publications in MAG than their portion of the node and edge counts may suggest.

Figure 2*: Information density represented as entropy in nits, and saliency for each hop in the CORD-19 closure graph expansion. As a reference, the entropy of the entire MAG dataset is 16 nits*

To understand how relevant the articles are to the CORD-19 throughout the hops towards the closure convergence, Figure 3 shows the average “embeddedness”, as defined in [23], of the articles, namely, the average ratio of citations received from within and outside of CORD-19C for articles encountered at each hop. The embeddedness of CORD-19 is 71.4% and articles cited up to the inflection point have the average embeddedness above 64%. After the inflection point, the embeddedness monotonically decreases, finally reaching 19% at the closure. This observation is consistent with the explanations offered by various models unified under the Discrete Choice theory [20] that scholars use topical closeness as a criterion in choosing articles to cite.

Figure 3*: Embeddedness, as defined in* [23]*, of the closure graph expansion per* hop.

To further verify this observation, the distribution of article fields of study, using the algorithm described in [27], are shown in Figure 4. Throughout the citation hopping process, the fields of biology, medicine, and chemistry dominate, with the portion of these three fields accounting for 50.4%, 38.0%, and 3.9% of the articles at CORD-19. By the inflection point at CORD-19I (hop 3), articles in medicine, at 28.3%, have overtaken biology at 26.7%, with the portion of articles in chemistry grown to 17.9%. Taken together, these top three fields comprise 92.3% and 73.9% of the articles in CORD-19 and CORD-19I, respectively. Agreeing with the trend shown with the embeddedness measure (Figure 3), articles from outside of the three fields start to increase in volume after the inflection point, eventually reaching 55.10% at the closure CORD-19C.

Figure 4*: Distribution of the top three high level fields of study using MAG field stamping algorithm* [27] *on each expansion hop of* the closure graph.

# Results

The questions on how, where, who are conducting research and what relevant scientific knowledge is being explored for COVID-19 can reach dramatically different answers from CORD-19 alone and from the expanded datasets described above. Again, the analyses presented here are based on the April 17, 2020 snapshot of CORD-19 and the corresponding enclosure graph CORD-19E, the inflection closure CORD-19I, and the full closure CORD-19C. Updated data, synchronized to CORD-19 daily releases, are publicly available at a GitHub repository at <https://aka.ms/magcord19mapping> and as a REST API, called Project Academic Knowledge with details available at <https://aka.ms/maservices>.

## Age Distributions of the COVID-19 Literature

CORD-19 has a strong bias towards newer articles, while its 3 expanded datasets capture better how the current research is built on knowledge discovered in the years past. Figure 5 illustrates the article age distributions in these 4 collections by showing the percentage of articles versus their publication years. Note that all areas under the curve are normalized to 100% even though their volumes are orders of magnitude apart (cf. Figure 1). Specifically, in CORD-19 where articles are dated back to the discovery of coronavirus in late 1960’s, a whopping 12% of the articles are published in 2020. A steep jump in article counts can be seen in year 2003, corresponding to the major outbreak of SARS that is also caused by a novel coronavirus. In contrast, the expanded sets all show a gradual rise in articles from the decades before 2010, suggesting the scientific knowledge contributing to COVID-19 research today is accumulated over a long period of time. Based on the two closure graphs, CORD-19I or CORD-19C that bear remarkable identical results, the body of research literature of year 2020 still account for 0.02% even with the flurry of the publications on the subject in the first few months of year. CORD-19E, which includes articles citing CORD-19, contains more articles in the recent years than the closure graphs. Still, CORD-19E has less than 2% of articles published in year 2020 and shows the literature of the past decades accounts for a larger presence than those in recent years as suggested by CORD-19.

Figure 5: *Distribution of articles in percent per year for CORD-19 (seed), CORD-19I (inflection), CORD-19C (closure), and the enclosure graph CORD-19E generated by the bi-directional citation expansion. Callouts are percentages for CORD-19 and CORD-19C for the years 2002, 2012, and 2019-2020, corresponding to the SARS, MERS, and COVID-19 outbreaks, respectively.*

Aside from analyses based on article counts alone, Figure 6 shows the publication year distribution where the articles are further weighted by their respective importance. Again, while CORD-19 strongly emphasizes the importance of recent articles, the expanded datasets recognize more the contributions from the past decades. Specifically, both the citation counts (shown as dotted lines) and the saliencies are first computed as measurements for article importance before being aggregated for each publication year in Figure 6. Here, the age bias in citations, reported to be 7 to 10 years as first noted in [26], manifests itself as the visibly lower citation counts for articles from the most recent decade. Their saliencies, designed to mitigate the age bias somewhat, shift the distribution towards more recent years. All expanded datasets indicate the articles in the decade before 2018 are more likely to be considered as important in the near future, as opposed to the CORD-19 results that suggest articles published in 2020 have an outsized probability, 13.2%, being cited against other CORD-19 articles, e.g., 3.9% for those published in 2019 and 4.6% for 2018.

Figure 6: *Distribution of article importance as measured by citation counts (dotted lines) and saliencies (solid lines) for CORD-19 and the 3 expanded datasets.*

## Journal Composition of the COVID-19 Literature

In addition to aggregating articles by their publication year, publication journals are another dimension that shows the analytical results based on CORD-19 and its the expanded datasets are very different. Note that, to help accelerate research on COVID-19, many but not all journals have made full texts of related articles available as part of the CORD-19 dataset. These articles are selected by the publishers through the PubMed Central with their editorial judgments [5]. To inspect the potential selection biases, Figure 7 first compares the number of articles that are part of the CORD-19 dataset from top journals to the number of articles from those same journals that are part of CORD-19E. The article counts being presented in the logarithmic scale, the height difference for each journal corresponds to the coverage, or the percentage of CORD-19E articles being sampled into CORD-19. As observed, the CORD-19 collection is several orders of magnitude smaller than that of CORD-19E in terms of article coverage with some journals higher than others. The difference represents an opportunity for these journals to contribute for increasing the number of freely available articles in the CORD-19 dataset. Similarly, the publisher contribution opportunities can also be computed by comparing the number of articles in the inflection and final closure graph CORD-19I and CORD-19C, as seen in Figure 8. As can be seen with the closure graphs, CORD-19 has considerably uneven coverage among its publication venues. Archival services such as bioRxiv and medRxiv have close to perfect representation in CORD-19, while journal articles see coverage varying significantly from one to another.

Figure 7: *Article counts (primary vertical axis in log scale) in CORD-19 and CORD-19E from the top 15 journals ranked by saliencies based on CORD-19 articles. An uneven coverage among journals in CORD-19 can be seen against the CORD-19E baseline, with medRxiv having the highest coverage in that almost all CORD-19E articles there are already included in CORD-19. Additionally, the journal saliencies computed from CORD-19E are overlaid as the grey line along the secondary vertical axis. The journal rankings derived from the two datasets show dramatic* differences.

Figure 8: *Article counts in CORD-19 versus CORD-19C and CORD-19I from top 15 journals ranked by saliencies based on CORD-19C articles. As in*

*Figure 7, the data show uneven coverage of journals in CORD-19, especially for highly ranked journals such as Science, NEJM, and Journal of ACS. The journal saliencies based on CORD-19I are shown as the yellow line along the secondary vertical axis, whose monotonical decrease indicates the journal rankings derived from the partial closure are already in close agreement with those derived from the full closure*.

Aside from the quantities of articles being included, another way to assess the sampling efficacy of CORD-19 is to test if journals are represented proportionally to their impacts, for which an article-level impact indicator, namely, the saliency, is used to aggregate at the journal level so that the best practice as described in the San Francisco Declaration of Research Assessment [28] is honored. Specifically, the saliency of a journal is calculated with the formula $s_{J}(C)=\sum_{a\in C\cap J} s_{a}$, where $s_{J}, s_{a}$ denote the saliencies of the journal $J$ and the article $a$, respectively, whereas $C$, the documentation collection under which the journal impact is to be assessed. The formula clearly demonstrates how an article-level journal impact assessment is dependent upon the document collection used.

Figure 7 shows the rankings of journals, high to low from left to right, based on their CORD-19 saliencies (i.e., $C=$CORD-19), while their corresponding CORD-19E saliencies are also overlaid, showing how these journals would be ranked under the expanded set. The result highlights a dramatically different journal rankings between CORD-19 and the expanded set. Similarly in Figure 8 where the journal rankings are based on CORD-19I or CORD-19C, the results exhibit significant differences between CORD-19 and the expanded datasets, although the rankings from the expanded sets are in remarkable agreements with each other despite their significant difference in article counts (cf. Figure 1). A notable observation is the top journal according to CORD-19 is a specialty journal on virology, while the expanded sets rank highly the general yet prestigious journals such as Nature, Science, and PNAS. Particularly, the lack of sufficient coverage of journal Science contributes to the journal not ranked as high based on CORD-19 (Figure 7) as based on either CORD-19I or CORD-19C (Figure 8).

## Research by Geo-Political Regions

The geographical data in MAG also affords the study on where the research is conducted based on geo-political region clustered broadly by continents. Articles are mapped to authors’ affiliations which are located in each of geo-political regions shown in *Figure 9*. Each article is attributed equally to all regions represented by its affiliations, as is the case when an author has multiple affiliations. Therefore, an article may be counted more than once depending on the number of regions its authors are affiliated with. For ease of visualization, only articles from the year 1990 onwards are used for this analysis. Articles in the CORD-19 dataset are primarily authored in the Americas (~40%), followed by Asia and then Europe taking a nearly equal share of ~27%, each. The number of articles per year in CORD-19C peaks in 2013, likely due to the age bias of 7 to 10 years in the citation network first observed by Price [26]. Regardless, CORD-19C shows a noticeable distinction between regions, with Americas accounting for 39% of total articles, alongside Europe with 34%, and Asia with 22%, respectively. It is evident from both collections that research contributions from Asia are increasing over time, not just specific to COVID-19 research but even within the closure that contains research across many disciplines.

Figure 9: *Article counts per year by continents for CORD-19 and the 3 expanded datasets*.

Figure 10 shows the percentage of articles per year contributed by each continent. A spike in articles from Asia is observed in the CORD-19 collection for the years, 2003 and 2020, corresponding to the SARS and COVID-19 outbreaks, respectively. As the graph is expanded (enclosure, inflection, and closure) to include other relevant literature, the 2003 spike is smoothed out and no longer observed, but additional focus is brought on the fact that, while the Americas have historically produced the lion’s share of articles, contributions from Asia have continued to increase steadily, signifying a persistent trend.

Figure 10*: Geo-political distribution of articles by year for CORD-19 and the 3 expanded datasets.*

Unlike the case of journal rankings, the qualitative analyses on geolocational distributions based on CORD-19 and its expanded sets are largely consistent with one another. While the distributions in CORD-19 appear ragged in Figure 10, the results from the expanded sets are much smoother as expected per their design to be statistically more stable. Once again, the results from the partial closure CORD-19I are remarkably close to the full closure CORD-19C, even though the former is a fraction in size of the latter (cf. Figure 1).

## Author Team Sizes

Figure 11 shows the distribution of team sizes that are estimated by counting the number of authors per article for each dataset (seed, enclosure, inflection and the full closure). Again, for presentation simplicity, only data after 1990 are shown. There is a sustained increase in the team size over years, observed in each of the four graphs, until 2019 where it dips. This can also be verified by the median and the average team sizes in all datasets, also shown in Figure 11. Again, CORD-19 can be seen to have a more ragged distribution that is smoothed out in the expanded datasets, and the qualitative trends agree with one another.

Figure 11*: Team size distribution of authoring teams, by year, across CORD-19 and its 3 closure graph expansions.*

Quantitatively, though, the team size analyses are more conditioned upon the manners in which these datasets are constructed. For example, the reduction in team sizes is less pronounced in CORD-19E than either CORD-19I or CORD-19C. In fact, the distribution, the average and median team sizes suggest a continuing trend of larger teams, in line with the observations made by [29]. This result can be attributed to the fact that CORD-19E contains both citing and cited articles, where the partial or full closure graphs only consider the cited articles and hence their compositions are more susceptible to the inherent under-representation of newer articles in the citation network [26]. However, a comparison between CORD-19I and CORD-19C indicates that the partial closure contains a greater portion of articles from larger teams, as can be seen from the team size distributions and the median team size statistics. This observation suggests articles from larger teams are encountered earlier in the network traversal process, namely, they are cited more directly and hence easier to have their impacts observed. Paradoxically, the average team size statistics, especially in recent three years, exhibit higher numbers in CORD-19C than CORD-19I. A quick check into the datasets reveals this is an outcome of a few articles with extraordinarily large team sizes that are encountered in later hops and highly skew the distribution. The fact of their late inclusion into CORD-19C provides a counterexample to the notion that articles from larger teams are monotonically more likely to be cited. In the meantime, this case highlights the sensitivity of the average team size statistic to the skewness caused by a handful yet highly irregular data points, and is an example of the relative resilience of using median as a measure.

## Interdisciplinary Research

The journal distributions, especially the wide varieties of specialized journals in Figure 7 and Figure 8, indicates a strong interdisciplinary interaction among fields related to COVID-19 research. As journals are merely a proxy of the fields of study, the cross-topical citations are further examined using the article-level field of study labels provided in MAG that utilizes natural language features and publication metadata to extract the topics of an article [27]. Figure 12 shows the distribution of citations from one field to another, or interdisciplinary references, for the top three fields of study within CORD-19 and the three expanded datasets. Because citations are directed, each cell within each matrix in this figure represents the percentage of references going from articles stamped with a field in the rows to an article stamped with a field in the columns of the matrix. For example, in the CORD-19 matrix we can see that 42.5% of articles labeled with “Chemistry” cite articles labeled with “Biology”. Each topic row of each matrix corresponds to all articles labeled with that topic and therefore each row percentage adds up to 100%. Data in Figure 12 presents the following observations:

1. The citation distribution coming from the top three fields starts out heavily skewed in favor of Biology and Medicine but evens out considerably in later hops of the expansion, i.e., the inflection and closure graphs.
2. The ratio of citations coming from the top three fields compared to other fields gets lower in later hops, starting at nearly 95:5 in CORD-19, to about 58:42 in CORD-19C. This clearly indicates a broadening of content with respect to fields of study as the collection is expanded. Interestingly, the citation ratio between top three fields to others is still 78:22 at the inflection point, which means that the content at the inflection hop remains domain specific to the domains dominating the seed collection.
3. There is a very strong intra-disciplinary citation signal as the mass of citations collects along the diagonals in each matrix in the figure. Article citations in each field of study become increasingly insular as more articles are added to the collection.

Figure 12*: Topic distribution and interdisciplinary citation distribution for the top 3 high level topics in closure graph expansions CORD-19, CORD-19E, CORD-19I, and CORD-19C. The value in each cell is the percentage of citations going from the field of study on the left (row) to the field of study on the bottom (column). The bar over each matrix shows the percentage of citations coming from each field*

The claim that expanding CORD-19 to larger datasets can result in more holistic views on topic diversity – as well as more interdisciplinary citations – is best seen by further analyzing the citations coming from articles labeled as “Chemistry” articles in Figure 12. Initially, about 53% of the Chemistry articles included in the original CORD-19 dataset cite works in Medicine and/or Biology. This would suggest that, from the vast collection of human knowledge about the broad subject of Chemistry, most Chemistry articles included in the CORD-19 dataset are relevant to problem at hand. But as we get further along the expansion, the percentage of Chemistry articles citing Medicine or Biology continues to decrease until finally, reaching the closure point (CORD-19C), only 20% of Chemistry articles included in the collection cite Medicine/Biology articles while the rest cite other disciplines, indicating that a more diverse corpus of Chemistry knowledge is now included in the collection.

However, the topical bias in CORD-19 (shown in Figure 4) lurches behind such analyses in a dramatic way. If the interdisciplinary research were to study with the incoming citations, the lack of proper coverage of Chemistry articles would lead CORD-19 to suggest that Chemistry articles are cited more by articles in Biology than in the same field. Figure 13 illustrates this result with a different style of visualization to highlight the discrepancies in conclusions CORD-19 and its expanded sets can lead to. Again, the topical bias in CORD-19 can be somewhat mitigated with citations, even in CORD-19E where a single step of expansion is used. Similarly, the more elaborated results with CORD-19C show the within-field self-citation for Chemistry is no less uncommon, more than 60%, and are already consistent with the results derived from the much smaller dataset CORD-19I.

*Figure 13: Percentage distributions of citations received for the top 3 fields in CORD-19 and its expanded sets.* In contrast to Figure 12 that computes the distributions based on the outgoing references, the statistics based on incoming citations shown here are distorted more seriously by the lack of coverage of Chemistry articles in CORD-19: e.g., one would conclude Chemistry articles are cited more by articles of Biology than of the same field that cannot be corroborated by the expanded sets.

## Full Text Availability

A key objective for CORD-19 is to make the full text of COVID-19 research articles widely available for advanced analyses which, indeed, has received positive responses from many publishers. However, many of the publisher licenses granted to CORD-19 expire on the date when the pandemic is declared over by WHO. Although there are few indications that the date is fast approaching, it does raise a concern on the longevity of the dataset. To estimate the impacts of the special open text license by the publishers,

Figure 14 shows the percentage of open access (OA) articles in CORD-19 as well as its expanded datasets using the information provided by MAG. Specifically, an article is counted as OA if any of the following conditions are met:

1. The article has a version found on an archival service such as bioRxiv, medRxiv.
2. The article is included in the OA repository CORE at core.ac.uk.
3. A PDF version of the article, excluding its DOI destination, can be found in the Bing web index, e.g., from authors’ homepages or institutional websites that host author accepted manuscripts (AAM).

*Figure 14: Portion of articles with full text contents available in* MAG

By excluding versions from the publishers, the tabulation is a lower bound estimate of OA articles. As can be seen, there is generally an encouraging trend of increasing OA adoption over the past decades while recent articles, many still under embargo, see a lower rate. The sudden reversal of OA rate for year 2020, prominently visible across all four datasets, corresponds to the pandemic induced OA practices, either through the use of archival services or institutional websites. Note that this is one of the few examples where CORD-19I results are markedly different from CORD-19C where the latter does not show a drop in OA rate until 2019 and the former, in agreement with other datasets, shows the lowering OA rates starting in year 2015.

# Discussion

As the societies are fast moving to evidence-based decision making processes, often powered by large datasets whose qualities are not easy to manually inspect, the disciplines to understand the potential biases in the datasets and the operations on them have become ever more critical. For scholarly data, this work demonstrates how article citations from the scientists themselves can be leveraged by expanding the seed collection either to an enclosure set that contains articles cited or citing the seeds, or to a full or partial closure that traces the lineage that the seed articles derive their knowledge from. In the case of CORD-19, the expanded sets are all able to provide a much smoother picture in describing when the science of COVID-19 was first reported and consequently refined upon, who and where the research activities are conducted, and how scientists around the globe and from different fields are collaborating. Statistically speaking, the smoothed results from the expanded datasets indicate the sampling and observation errors in the creation of CORD-19 are more likely averaged out in the larger datasets. Most excitingly, even the enclosure CORD-19E can quickly include marginally related publications, the closure graph approach offers an effective alternative. Remarkably, the inflection closure CORD-19I can already accurately describe the trends and properties in the full closure CORD-19C, although the former is much quicker to get and is only a fraction in size of the latter.

The distinct portions of article ages in CORD-19 and its expanded sets may suggest the publishers use a very different notion in contributing their articles than the objective of enabling artificial intelligence (AI) agents to perform question answering tasks. By including more recent articles, CORD-19 dataset appears to be suitable for information retrieval applications that present contents for human experts that can draw domain knowledge from their training to fill in the background information not included in the collection. For machines to achieve the similar performance on par with human experts by having the equal background knowledge in reasoning with the facts and inferencing the plausible answers, it appears that the expanded datasets are more suitable for training AI agents. Particularly, the inflection closure CORD-19I seems to be a reasonable dataset as it strikes a balance in topical relevance, the manageable size, and the coverage of the prior arts.

Indeed, it is a surprise that the analytical results derived from the partial closure CORD-19I and the full closure CORD-19C are very close, not only in article ages but also in the analyses ranging from journal rankings to cross-disciplinary citation patterns. All these observations reinforce the notion that the inflection closure has already captured properties of the full closure. This outcome lends support to the network theories unified under the Discrete Choice model that scholars predominantly cite relevant articles that are widely considered as impactful in their fields. The empirical results suggest they can be tracked down by traversing the citation network with only a few hops. This suggests a robust methodology for systematically expanding the corpus of relevant knowledge for COVID-19 from the seed CORD-19, eventually reaching a corpus size that is two orders of magnitude larger. This method may be difficult and perhaps prohibitively expensive for publishers to implement in a short timeframe. To mitigate this problem, a sound approach would instead be to incrementally make new articles available by following the intermediate hops of the closure graph. This would allow publishers to first add articles directly related to COVID-19 in the short term (earlier hops) before opening up more general literature in the longer term (later hops), should the need arise. As the frequently and incrementally updated data are publicly available, this algorithm may result in a controlled release of articles, thereby amortizing the monetary overhead and information overload over time while still helping the broader research community. The analyses on journal coverage and their rankings, especially the differences between results from CORD-19 and the expanded datasets, may serve as an indicator to identify areas for improvement.

The citation behaviors manifest as the desired properties in CORD-19I and CORD-19C inspire a broader research question on the general applicability of the closure graph algorithm on other types of networks. After all, the theories behind preferential attachments and utility motivated choice of connection, which correspond to scholars citing already highly cited and relevant articles, can also be applied to explain the formation of a wide variety of networks. For instance, it might be worthwhile to test the efficacy of using the closure graph algorithm as a candidate for the community detection problem by starting with a seed collection of people in a social network and compute the full or partial closure based on their connections. In fact, MAG may already be a good dataset because it captures vital relationships among scholars, such as who have collaborated, followed, or critiqued on each other’s work. Testing the algorithm more broadly may also shed lights on a network property that this work has ignored, namely, the connection strengths on the network edges. For citation networks, the connection strength can be derived in many ways, for instance, from the content relevance between the contents in the citing and the cited articles, or the number of mentions to the cited article in the citing work. The latter is seen to play some roles in the estimation of saliency [25], but its significance in the closure graph has not been observed. As a result, the closure graph implementation reported here employs the most rudimentary breadth-first search algorithm in which all citations are treated as of equal strength. Preliminary studies indicate more sophisticated search methods, such as best-first or beam search, may be employed to arrive at alternatives more compact than CORD-19I with additional heuristics, e.g., applying a citation threshold to further filter articles in each hop, or limiting the expansion from each article with a beam width. However, as the effects of these heuristics seem to permeate through the datasets and impact the analytical results in a complicated manner, more studies are needed to tease apart the artifacts from the genuine core properties of the data.

The premise of this work is based on the widely accepted observation since [26] that the citation networks contain the largest possible, crowdsourced, and peer reviewed judgments on publications and thus can serve as a point of reference to evaluate the biases in a subsampled collection such as CORD-19. The validity of this and any methodologies built upon the citation networks, however, is predicated upon the citation behaviors not systematically compromised by inadvertent biases. In practice, however, not all citations are observable, especially for those made in the books or research articles whose contents, including the bibliography, are inaccessible publicly. As these unobserved citations are often characterized by systematic factors (e.g., publishers not sharing bibliographic information), they effectively introduce a form of publication bias into the citation-based methodology where research impacts recognized in certain types of publications are not included properly. MAG offers a potential mitigation against this kind of bias by inferring as missing links the “related” articles based on their content similarities and the co-citation patterns [25]. Missing link prediction, by all means, is far from a solved problem and indeed is a challenging research topic for which a community-driven open benchmark exists [30]. Although not studied in this work, how intelligence inference can potentially alleviate the problem of unobserved citations may prove essential in this and other citation network studies.

At the time of writing, more than 1000 contributions have been made to Kaggle based on the mirrored CORD-19 dataset [4]. Most of the contributions, including many based on clustering the articles to answers some of the questions posed by WH/OSTP and WHO, appear to have taken CORD-19 as is without explicitly mentioning the impacts on the accuracy of their answers based on biased data. It is our hope that this work can contribute to the general awareness of the implications of biased datasets and to the wide adoption of mitigating measures to debias the data.

# References

| [1] | S. P. Horbach, "Pandemic Publishing: Medical journals drastically speed up their publication process for Covid-19," *bioRxiv,* 2020. |
| --- | --- |
| [2] | Microsoft Research, "Changes to Microsoft Academic Services (MAS) During COVID-19," 7 April 2020. [Online]. Available: https://www.microsoft.com/en-us/research/project/academic/articles/changes-to-microsoft-academic-services-mas-during-covid-19/. |
| [3] | M. Hutson, "Artificial intelligence tools aim to tame the coronavirus literature," *Nature,* 9 June 2020. |
| [4] | [Online]. Available: https://www.kaggle.com/allen-institute-for-ai/CORD-19-research-challenge. |
| [5] | L. L. Wang, K. Lo, Y. Chandrasekhar, R. Reas, J. Yang, D. Eide, K. Funk, R. Kinney, Z. Liu, W. Merrill, P. Mooney, D. Murdick, D. Rishi, J. Sheehan, Z. Shen, B. Stilson, A. D. Wade, K. Wang, C. Wilhelm, B. Xie, D. Raymond, D. S. Weld, O. Etzioni and S. Kohlmeier, "CORD-19: The Covid-19 Open Research Dataset," *arXiv preprint arXiv:2004.10706,* 2020. |
| [6] | G. Colavizza, R. Costas, V. A. Traag, N. J. v. Eck, T. v. Leeuwen and L. Waltman, "A scientometric overview of CORD-19," *bioRxiv,* 2020. |
| [7] | K. Wang, Z. Shen, C. Huang, C.-H. Wu, Y. Dong and A. Kanakia, "Microsoft Academic Graph: When experts are not enough," *Quantitative Science Studies,* vol. 1, no. 1, pp. 396-413, 2020. |
| [8] | R. Sinatra, P. Deville, M. Szell, D. Wang and A. L. Barabási, "A century of physics," *Nature Physics,* vol. 11, no. 10, pp. 791-796, 2015. |
| [9] | E. Ntoutsi, P. Fafalios, U. Gadiraju, V. Iosifidis, W. Nejdl, M. Vidal, S. Ruggieri, F. Turini, S. Papadopoulos, E. Krasanakis, I. Kompatsiaris, K. Kinder‐Kurlanda, C. Wagner, F. Karimi, M. Fernandez, H. Alani, B. Berendt, T. Kruegel, C. Heinze, K. Broelemann, G. Kasneci, T. Tiropanis and S. Staab, "Bias in data‐driven artificial intelligence systems: An introductory survey," *Wiley Interdisciplinary Reviews-Data Mining and Knowledge Discovery,* vol. 10, no. 3, 2020. |
| [10] | A. Krizhevsky, I. Sutskever and G. E. Hinton, "ImageNet classification with deep convolutional neural networks," *Communications of The ACM,* vol. 60, no. 6, pp. 84-90, 2017. |
| [11] | O. Russakovsky, J. Deng, H. Su, J. Krause, S. Satheesh, S. Ma, Z. Huang, A. Karpathy, A. Khosla, M. Bernstein, A. C. Berg and L. Fei-Fei, "ImageNet Large Scale Visual Recognition Challenge," *International Journal of Computer Vision,* vol. 115, no. 3, pp. 211-252, 2015. |
| [12] | W. J. Cook, W. H. Cunningham, W. R. Pulleyblank and A. Schrijver, Combinatorial Optimization, 1997. |
| [13] | S. Redner, "How popular is your paper? An empirical study of the citation distribution," *European Physical Journal B,* vol. 4, no. 2, pp. 131-134, 1998. |
| [14] | A. L. Barabasi and R. Albert, "Emergence of Scaling in Random Networks," *Science,* vol. 286, no. 5439, pp. 509-512, 1999. |
| [15] | M. McPherson, L. Smith-Lovin and J. M. Cook, "Birds of a Feather: Homophily in Social Networks," *Review of Sociology,* vol. 27, no. 1, pp. 415-444, 2001. |
| [16] | G. Caldarelli, A. Capocci, P. D. L. Rios and M. A. Muñoz, "Scale-Free networks from varying vertex intrinsic fitness," *Physical Review Letters,* vol. 89, no. 25, p. 258702, 2002. |
| [17] | J. Leskovec, J. Kleinberg and C. Faloutsos, "Graph evolution: Densification and shrinking diameters," *ACM Transactions on Knowledge Discovery From Data,* vol. 1, no. 1, p. 2, 2007. |
| [18] | A. D. Broido and A. Clauset, "Scale-free networks are rare.," *Nature Communications,* vol. 10, no. 1, p. 1017, 2019. |
| [19] | P. Holme, "Rare and everywhere: Perspectives on scale-free networks.," *Nature Communications,* vol. 10, no. 1, p. 1016, 2019. |
| [20] | J. Overgoor, A. Benson and J. Ugander, "Choosing to Grow a Graph: Modeling Network Formation as Discrete Choice," in *The World Wide Web Conference on* , 2019. |
| [21] | M. Rosvall and C. T. Bergstrom, "Maps of random walks on complex networks reveal community structure," *Proceedings of the National Academy of Sciences of the United States of America,* vol. 105, no. 4, pp. 1118-1123, 2008. |
| [22] | M. Girvan and M. E. J. Newman, "Community structure in social and biological networks," *Proceedings of the National Academy of Sciences of the United States of America,* vol. 99, no. 12, pp. 7821-7826, 2002. |
| [23] | S. Fortunato and D. Hric, "Community detection in networks: A user guide," *Physics Reports,* vol. 659, pp. 1-44, 2016. |
| [24] | A. Sinha, Z. Shen, Y. Song, H. Ma, D. Eide, B.-J. (. Hsu and K. Wang, "An Overview of Microsoft Academic Service (MAS) and Applications," in *Proceedings of the 24th International Conference on World Wide Web*, 2015. |
| [25] | K. Wang, Z. Shen, C. Huang, C.-H. Wu, D. Eide, Y. Dong, J. Qian, A. Kanakia, A. Chen and R. Rogahn, "A Review of Microsoft Academic Services for Science of Science Studies," *Frontiers in Big Data,* vol. 2, 2019. |
| [26] | D. J. d. S. Price, "Networks of Science Papers," *Science,* vol. 149, no. 3683, pp. 510-515, 1965. |
| [27] | Z. Shen, H. Ma and K. Wang, "A web-scale system for scientific knowledge exploration," in *ACL 2018: 56th Annual Meeting of the Association for Computational Linguistics*, 2018. |
| [28] | Annual Meeting of the American Society for Cell Biology, "DORA," San Francisco, 2012. |
| [29] | Y. Dong, H. Ma, Z. Shen and K. Wang, "A century of science: globalization of scientific collaborations, citations, and innovations," in *Proceedings of the 23rd ACM SIGKDD International Conference on Knowledge Discovery and Data Mining*, Halifax, Canada, 2017. |
| [30] | W. Hu, M. Fey, M. Zitnik, Y. Dong, H. Ren, B. Liu, M. Catasta and J. Leskovec, "Open Graph Benchmark: Datasets for Machine Learning on Graphs," *arXiv: Learning,* 2020. |

# Figures and Tables

| Figure 1: Accumulated paper and citation counts per hop of the closure graph expansion. The callouts are article counts for CORD-19 (seed), CORD-19I (hop 3), and CORD-19C (hop 11). |
| --- |
| Figure 2: Information density represented as entropy in nits, and saliency for each hop in the CORD-19 closure graph expansion. As a reference, the entropy of the entire MAG dataset is 16 nits. |
| Figure 3: Embeddedness, as defined in [23], of the closure graph expansion per hop. |
| Figure 4: Distribution of the top three high level fields of study using MAG field stamping algorithm [27] on each expansion hop of the closure graph. |
| Figure 5: Distribution of articles in percent per year for CORD-19 (seed), CORD-19I (inflection), CORD-19C (closure), and the enclosure graph CORD-19E generated by the bi-directional citation expansion. Callouts are percentages for CORD-19 and CORD-19C for the years 2002, 2012, and 2019-2020, corresponding to the SARS, MERS, and COVID-19 outbreaks, respectively. |
| Figure 6: Distribution of article importance as measured by citation counts (dotted lines) and saliencies (solid lines) for CORD-19 and the 3 expanded datasets.  Figure 7: Article counts (primary vertical axis in log scale) in CORD-19 and CORD-19E from the top 15 journals ranked by saliencies based on CORD-19 articles. An uneven coverage among journals in CORD-19 can be seen against the CORD-19E baseline, with medRxiv having the highest coverage in that almost all CORD-19E articles there are already included in CORD-19. Additionally, the journal saliencies computed from CORD-19E are overlaid as the grey line along the secondary vertical axis. The journal rankings derived from the two datasets show dramatic differences. |

| Figure 8: Article counts in CORD-19 versus CORD-19C and CORD-19I from top 15 journals ranked by saliencies based on CORD-19C articles. As in  Figure 7, the data show uneven coverage of journals in CORD-19, especially for highly ranked journals such as Science, NEJM, and Journal of ACS. The journal saliencies based on CORD-19I are shown as the yellow line along the secondary vertical axis, whose monotonical decrease indicates the journal rankings derived from the partial closure are already in close agreement with those derived from the full closure. |
| --- |

| *Figure 9*: Article counts per year by continents for CORD-19 and the 3 expanded datasets. |
| --- |

| Figure 10: Geo-political distribution of articles by year for CORD-19 and the 3 expanded datasets. |
| --- |

| *Figure 11:* Team size distribution of authoring teams, by year, across CORD-19 and its 3 closure graph expansions. |
| --- |

|  | |
| --- | --- |
| Figure 12: Topic distribution and interdisciplinary citation distribution for the top 3 high level topics in closure graph expansions CORD-19, CORD-19E, CORD-19I, and CORD-19C. The value in each cell is the percentage of citations going from the field of study on the left (row) to the field of study on the bottom (column). The bar over each matrix shows the percentage of citations coming from each field.  Figure 13: Percentage distributions of citations received for the top 3 fields in CORD-19 and its expanded sets. In contrast to Figure 12 that computes the distributions based on the outgoing references, the statistics based on incoming citations shown here are distorted more seriously by the lack of coverage of Chemistry articles in CORD-19: e.g., one would conclude Chemistry articles are cited more by articles of Biology than of the same field that cannot be corroborated by the expanded sets. |  |

Figure 14: Portion of articles with full text contents available in MAG

=-]]

‘kp[]
